# Non-Centered Spike-Triggered Covariance Analysis Reveals Neurotrophin-3 as a Developmental Regulator of Receptive Field Properties of ON-OFF Retinal Ganglion Cells

**DOI:** 10.1371/journal.pcbi.1000967

**Published:** 2010-10-21

**Authors:** Donald R. Cantrell, Jianhua Cang, John B. Troy, Xiaorong Liu

**Affiliations:** 1 Interdepartmental Neuroscience Program, Northwestern University, Evanston, Illinois, United States of America; 2 Department of Biomedical Engineering, Northwestern University, Evanston, Illinois, United States of America; 3 Department of Neurobiology and Physiology, Northwestern University, Evanston, Illinois, United States of America; Université Paris Descartes, Centre National de la Recherche Scientifique, France

## Abstract

The functional separation of ON and OFF pathways, one of the fundamental features of the visual system, starts in the retina. During postnatal development, some retinal ganglion cells (RGCs) whose dendrites arborize in both ON and OFF sublaminae of the inner plexiform layer transform into RGCs with dendrites that monostratify in either the ON or OFF sublamina, acquiring final dendritic morphology in a subtype-dependent manner. Little is known about how the receptive field (RF) properties of ON, OFF, and ON-OFF RGCs mature during this time because of the lack of a reliable and efficient method to classify RGCs into these subtypes. To address this deficiency, we developed an innovative variant of Spike Triggered Covariance (STC) analysis, which we term Spike Triggered Covariance – Non-Centered (STC-NC) analysis. Using a multi-electrode array (MEA), we recorded the responses of a large population of mouse RGCs to a Gaussian white noise stimulus. As expected, the Spike-Triggered Average (STA) fails to identify responses driven by symmetric static nonlinearities such as those that underlie ON-OFF center RGC behavior. The STC-NC technique, in contrast, provides an efficient means to identify ON-OFF responses and quantify their RF center sizes accurately. Using this new tool, we find that RGCs gradually develop sensitivity to focal stimulation after eye opening, that the percentage of ON-OFF center cells decreases with age, and that RF centers of ON and ON-OFF cells become smaller. Importantly, we demonstrate for the first time that neurotrophin-3 (NT-3) regulates the development of physiological properties of ON-OFF center RGCs. Overexpression of NT-3 leads to the precocious maturation of RGC responsiveness and accelerates the developmental decrease of RF center size in ON-OFF cells. In summary, our study introduces STC-NC analysis which successfully identifies subtype RGCs and demonstrates how RF development relates to a neurotrophic driver in the retina.

## Author Summary

The developmental separation of ON and OFF pathways is one of the fundamental features of the visual system. In the mouse retina, some bi-stratified ON-OFF RGCs are refined into mono-stratified ON or OFF RGCs during the first postnatal month. However, the process by which the RGCs' physiological receptive field properties mature remains incompletely characterized, mainly due to the lack of a reliable and efficient method to classify RGCs into different subtypes. Here we have developed an innovative analysis, Spike Triggered Covariance – Non-Centered (STC-NC), and demonstrated that this technique can accurately characterize the receptive field properties of ON, OFF and ON-OFF center cells. We show that, in wildtype mouse, RGCs gradually develop sensitivity to focal stimulation after eye opening, and the development of ON-OFF receptive field center properties correlates well with their dendritic laminar refinement. Furthermore, overexpression of NT-3 accelerates the developmental decrease of receptive field center size in ON-OFF cells. Our study is the first to establish the STC-NC analysis which can successfully identify ON-OFF subtype RGCs and to demonstrate how receptive field development relates to a neurotrophic driver in the retina.

## Introduction

Many studies have investigated the segregation of ON and OFF pathways in the retina during postnatal development, and much is known about the structural maturation of different subtypes of retinal ganglion cells (RGCs) [1], [2]. For example, based upon the sublamina in which RGC dendrites arborize in the inner plexiform layer (IPL), RGCs can be classified into three subtypes: ON, OFF, and ON-OFF, which presumably respond to light onset, light offset, and both [3]–[5]. RGCs acquire their final dendritic branching pattern and territories in a subtype-dependent manner [6]–[9]. In the mouse, RGC dendritic arbors ramify diffusely in the IPL shortly after birth and then undergo extensive laminar refinement [8]–[11]. Consequently, the fraction of bistratified RGCs decreases as they are converted into monostratified cells during the first postnatal month [8], [10]. While RGCs having dendrites monostratified in the ON sublamina continue to expand their dendritic field size by adding new branches following eye-opening, bistratified RGCs acquire their final morphology by the time of eye opening [8], [9].

Far less is known about how the development of the physiological properties of different RGC subtypes might correlate with their dendritic refinement during postnatal development. This is largely due to the lack of a reliable method to identify ON, OFF and ON-OFF center RGCs in the mouse. The full field flash stimulus is often used in visual experiments [11]–[13]; for example, Tian and Copenhagen (2003) showed that with this stimulus the number of RGCs with ON-OFF responses decreases after eye-opening. However, because full field flashes stimulate both the center and the surround of the receptive field (RF), responses evoked by this stimulus cannot be linked reliably to center-type. Furthermore, RF structure cannot be studied with full field flashes because of the spatially uniform nature of the stimulus. Spatiotemporal white noise [14] has become a quite commonly used high-dimensional visual stimulus to investigate the spatial extent and temporal properties of RFs. Visual neural responses to white noise are typically modeled with a linear filter followed by a static nonlinearity (the LN model) [15], [16]. The Spike-Triggered Average (STA), which is the mean visual stimulus that precedes a spike, can be calculated from a cell's response to white noise, and is often used as the linear filter in LN models of ON and OFF center RGCs [16]. The STA approach struggles however when responses depend on symmetric static nonlinearities [17], such as might be expected for an RGC with an ON-OFF center. For example, the STA technique has been applied to classify RGCs into different ON or OFF subtypes, but not the ON-OFF subtype [18]. In such situations, spike-triggered covariance (STC) analysis, which identifies multiple relevant linear filters, provides a better analytical approach [19]–[21]. However, full STC analysis can be cumbersome and data-hungry, therefore we develop in the current study a Non-Centered Spike-Triggered Covariance (STC-NC) analysis which maintains the simplicity of a single filter analysis but is capable of characterizing different RGC subtypes, including the ON-OFF center variety.

The molecular players involved in RGC dendritic development have begun to be identified. Both brain-derived neurotrophic factor (BDNF) and Neurotrophin-3 (NT-3) have been shown to modulate RGC laminar refinement and dendritic branching in a subtype-specific manner [8], [9]. We and others have shown that both brain-derived neurotrophic factor (BDNF) and Neurotrophin-3 (NT-3) modulate RGC laminar refinement and dendritic branching during postnatal development [8]–[10]. While both BDNF and NT-3 accelerate RGC laminar refinement, their effects on dendritic branching is cell-type specific [8], [9]. BDNF selectively promotes formation of new branches in monostratified, but not bistratified RGCs [8]. By contrast, NT-3 promotes the formation of more, but shorter, branches selectively in bistratified RGCs, resulting in smaller but more dense dendritic trees [9]. It is less clear how these neurotrophin-dependent dendritic structural changes of RGCs relate to refinement of function.

Here we have investigated the physiological development of RGC RF properties after eye-opening, and the regulation of this process by NT-3. We introduce and characterize the STC-NC analysis, which is an innovative variant of the STC method and able to accurately identify ON, OFF, and ON-OFF center character in RGCs. With this new method, we show that wildtype RGCs gradually develop sensitivity to focal stimulation after eye-opening. The percentage of ON-OFF center cells decreases with age, and the RF centers of ON and ON-OFF cells become smaller. Importantly, overexpression of NT-3 leads to a precocious maturation of RGC responsiveness and accelerates the developmental decrease of RF center size in ON-OFF cells.

## Results

### Neither full field-evoked responses nor the STA classifies RGC subtypes accurately

**Figure 1:**
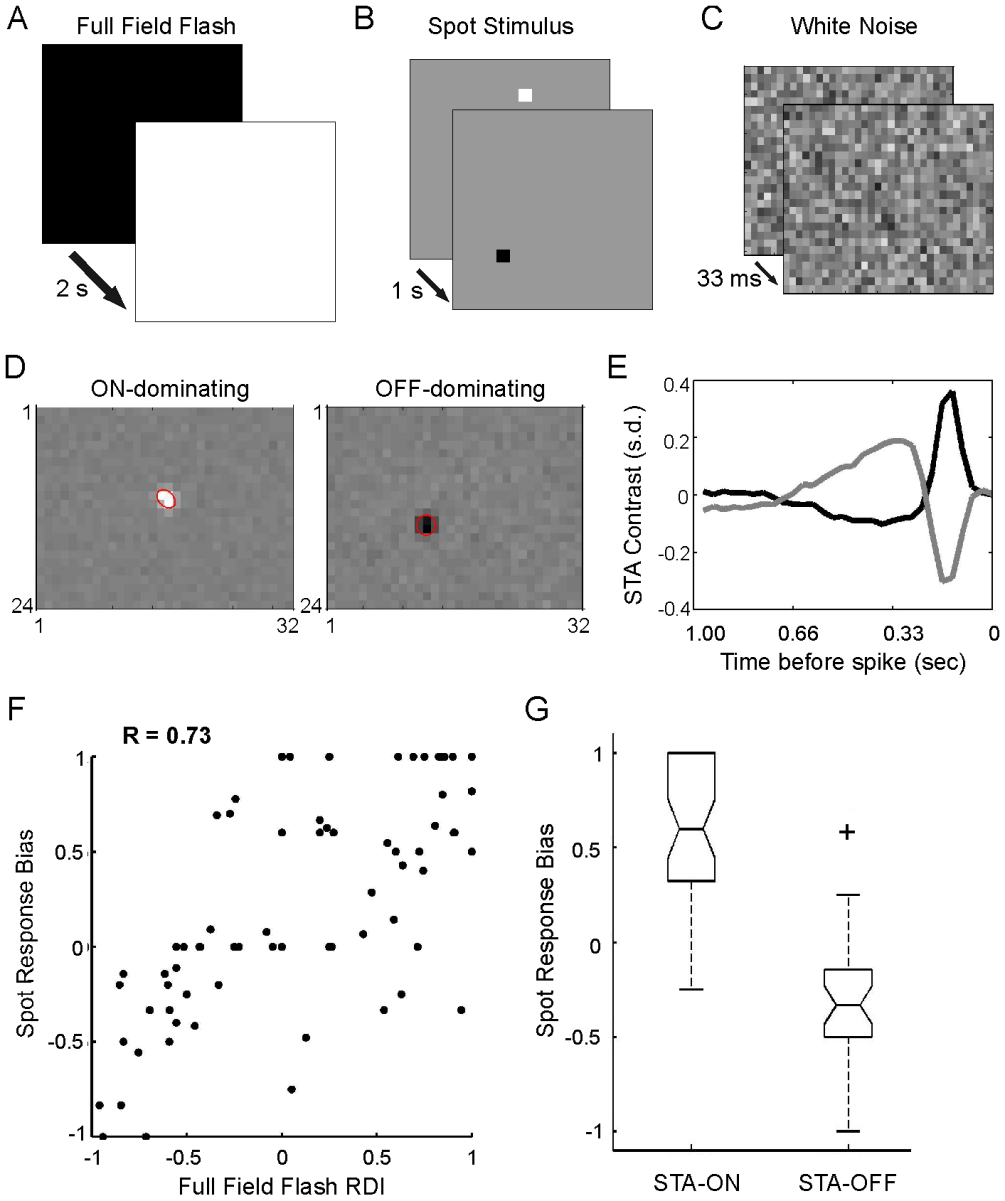
Comparison of cell-typing using responses to the full field flash, the spot, and the white noise stimuli. Schematics of the full field flash (A), spot (B), and white noise (C) stimuli used to characterize RGC RF centers. (D) Averaged single frames taken at the maximum (ON-dominating) or minimum (OFF-dominating) of the STA time course (E) illustrating their RF center locations and sizes. A 1-standard deviation (s.d.) Gaussian contour estimate of the RF center is overlaid in red. (E) STA time course for an ON-dominating cell (black) and an OFF-dominating cell (gray). (F) Scatter plot of the Spot Response Bias versus the Full Field Flash Response Dominance Index (RDI). The measures were correlated (R = 0.73), but there were many cells with discrepant classifications by the two measures, particularly for ON-OFF cells. (G) Boxplot of the distribution of the Spot Response Bias for ON and OFF STAs shows that many ON-OFF cells revealed through the spot stimulus were classified as ON or OFF cells by the STA. Horizontal box lines represent the lower quartile, median, and upper quartile values of the distribution. Notch represents the 95% confidence interval around the median. Whiskers (dashed lines) show the extent of the remaining data and the plus sign represents an extreme outlier.

Three types of visual stimuli, the full field flash, a spot stimulus, and a white-noise checkerboard stimulus, were applied to investigate RF properties of RGCs (Fig. 1). The full field flash stimulus (Fig. 1A) is easy to generate, and with high contrast it evokes robust responses [11], [22]. Classification through the Response Dominance Index (RDI, see Materials and Methods) identifies positive values with ON and negative values with OFF RGCs. Those cells which give robust responses and have an RDI near zero are conventionally classified as ON-OFF RGCs (Fig. 1F). However, as noted earlier, full field flash stimuli drive both center and surround RF components, making classification based on responses to this stimulus an inaccurate reflection of RGC center type. By contrast, a localized flashing spot only stimulates the RF center of an individual cell (Fig. 1B), and the Spot Response Bias can be calculated to identify ON, OFF and ON-OFF centers (Fig. 1F–G). However, this approach provides a time-consuming and inefficient way to identify RGCs when a microelectrode array system is used where the discharges of many cells can be recorded (and potentially stimulated) simultaneously.

Compared to the full field flash and the spot stimulus, a white-noise checkerboard stimulus (Fig. 1C) permits focal stimulation of many RGC centers simultaneously. It thus combines the spatial localization advantage of spot stimulation with the advantage enjoyed by the full field flash stimulus of gathering data from a large population of cells at once. The STA is the standard measure employed for identifying RF center responses with checkerboard stimuli. As expected, STAs possessed large deviations from the mean luminance (2 cd m^−2^) only in a small spatially confined region of the display, the center of the neuron's RF, and tapered to near mean luminance away from the RF center, indicating that stimulus perturbations at these distant locations were uncorrelated with spikes (Fig. 1D). To determine the center size, a bivariate Gaussian was fit to the single STA frame of maximal contrast and the area within the ellipse formed by the fitted Gaussian's 1σ contour used (Fig. 1D). For each frame of the STA, pixel contrast was averaged over the spatial STA indices located within the 1σ contour of the bivariate Gaussian, and these mean contrast intensities were plotted as a function of time prior to the spike (Fig. 1E). This time course, which is biphasic in structure for most cells and monophasic for a small number of cells, provides identification of ON and OFF character. However, a large population of bistratifying presumed ON-OFF center cells are present in the mouse retina [8], [11], [23], so some cells identified as ON or OFF center with the STA could well be ON-OFF center cells with unbalanced ON and OFF components. We therefore labeled cells either ON-dominating (instead of ON-center, see below) if the maximal positive contrast preceded the spike more closely than the maximal negative contrast, or OFF-dominating in the converse case.

We compared cell identification with the full field flash RDI, Spot Response Bias and the STA. We find that the full field flash RDI correlates positively with Spot Response Bias (R = 0.73, p<0.001), but in many cases the RDI failed to reveal the character of the RF center determined by the spot stimulus (Fig. 1F). RGCs with RDI values near zero, suggesting an ON-OFF response, ranged in Spot Bias from 1 (ON-center) to −1 (OFF-center, Fig. 1F). Similarly, correlation between STA values and Spot Response Bias is unsatisfactory; RGCs with both ON and OFF STA signatures exhibited a broad range of Spot Response Bias values (Fig. 1G). Cells possessing ON-OFF center character as determined by the spot stimulus were included in both ON and OFF STA classes (Fig. 1G). Clearly, there were many instances where the full field flash RDI and the STA measures failed to provide an accurate, or even coherent, classification of RGC cell-type, particularly for cells with ON-OFF character (also see Table S1 for a summary).

### A new computational method to identify ON-OFF center RGCs: Spike-Triggered Covariance - Non-Centered (STC-NC) analysis

To provide a more accurate characterization of RGC subtypes, including the ON-OFF center type, we employed white noise analysis techniques that were based on the second moment of the spike-triggered ensemble (STE), rather than upon its mean (i.e. STA). STC analysis proceeds by performing a principal component analysis (PCA) on the STE. PCA can be achieved by eigendecomposition of the covariance matrix, which generates eigenvectors that are then sorted by their eigenvalues to identify directions of large and small variance. Traditionally, PCA is performed by the eigendecomposition of the mean-centered covariance matrix. Mean centering is mathematically founded as it guarantees that the low-dimensional hyperplane created by the few principal components with greatest eigenvalues is the best fitting hyperplane for the high-dimensional data set in the mean square error sense [24]. Additionally, mean centering ensures that during dimensionality reduction by projection of the data onto the top principal components, maximal data variance is retained [24]. Because the retinal circuitry is divided into ON and OFF pathways, the polarity of the stimulus that is required to elicit a spike is of primary importance. The stimulus polarity is determined by the positive or negative deviation of the stimulus from mean luminance. For this reason, mean luminance, which is represented by the zero vector in our high dimensional stimulus space, is a critical reference point. To identify the single direction in stimulus space that maximizes the second moment of the STE about zero, we chose to perform eigendecomposition on a non-centered second moment matrix **M**:
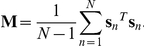



In the case of a non-centered moment matrix, the eigenvector with the greatest eigenvalue maximizes the second moment of the STE *around zero* – not the variance, which is the second moment *around the mean*. The hyperplane created by the eigenvectors with the greatest eigenvalues is the best-fitting hyperplane that passes *through the origin* [24], [25]. We term this technique STC-NC analysis.

**Figure 2:**
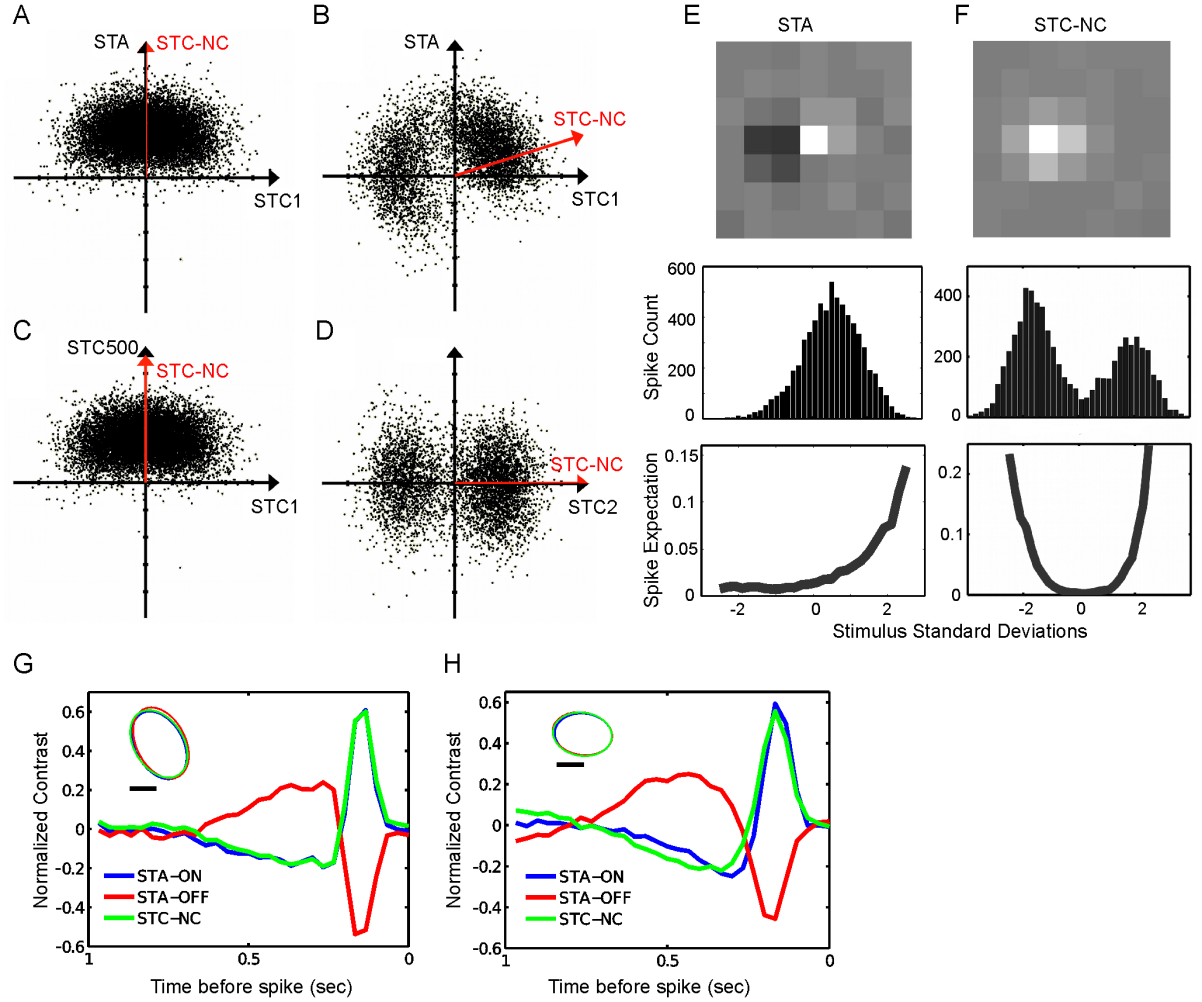
The linear filter discovered by non-centered spike triggered covariance (STC-NC) analysis aligns with the STA for ON or OFF cells and the high variance STC for ON-OFF cells. (A–B) Scatter plots of the STE projected onto the STA and the high variance STC (STC1) for an ON cell (A) and an ON-OFF cell (B) shows that the STC-NC aligned extremely well with the STA for the ON cell, but not for the ON-OFF cell, in which the STC-NC clocks toward the STC1. The STA was projected out of the STE prior to computing the STC only in A–B to ensure that the STA was orthogonal to the discovered STC vectors. (C) Scatter plot of the STE against the high and low variance STC vectors for the same cell in (A). The STC-NC aligned better with the STA in panel A than the low variance STC (STC500). (D) Scatter plot of the STE against the two STC vectors of greatest variance for the same cell in (B). The STC-NC aligned extremely well with the STC of highest variance. The length of the STC-NC vector (red) corresponded to the degree to which it projects onto the 2D plane. (E–F) Spatial frame of maximal contrast, 1D projection of the STE, and recovered static nonlinearity using the STA (E) and STC-NC (F, STC-NC bias = −0.11) for the ON-OFF cell from (B) and (D). The STA misidentified this cell as an ON cell and provided a poorly defined RF. (G–H) Some ON-OFF cells possessed spatiotemporally overlapping and inverted ON and OFF RFs (G), and some possessed slower OFF RF filters (H). The STC-NC accurately captured the character of both the ON and OFF filters for the cell in panel G, but not in panel H. The insets show the spatial RF centers (Scale bar: 100 µm).

We compared the results of our new technique with those of conventional mean-centered STC analysis in Fig. 2 (A–D). These panels plot the full set of the STE, utilizing a geometrical representation of an M-dimensional stimulus space, where M is the number of pixel intensities in a spatiotemporal stimulus. In this space, each individual spatiotemporal stimulus, which is simply a vector of pixel intensities, can be represented by a single point. Choice of axes is particularly important because they are used to reduce the dimensions of the space for a more manageable presentation and analysis. The perpendicular axes of the space were determined by the conventional STC analysis, which was performed either with (Fig. 2A–B) or without (Fig. 2C–D) projecting the STA out of the STE [21], [26]. The red vector illustrates the direction of the STC-NC and its relationship to the STA and the STC principal components. The length of the STC-NC vector, compared to the length of the axes, is used to graphically represent the degree to which the M-dimensional vector projects into the 2D space shown.

For cells possessing strong ON-OFF character, STC analysis captured the most important stimulus dimension in the highest variance PC, when the STA was not projected out of the STE (Fig. 2D). The STC-NC closely matched the low variance PC (STC500) for cells of ON or OFF character (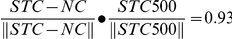), and matched the high variance PC (STC1) for cells of strong ON-OFF character (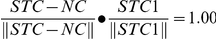, Fig. 2D). In this case, the STA was near orthogonal to this direction (
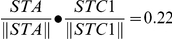
, Fig. 2D), showing that this measure captured little of this response-related variance. As a comparison, cells with ON or OFF character were often best described by the lowest variance PC. This PC aligned extremely well with the STA, as evidenced by the near unity normalized inner product of the STA with the low variance PC (
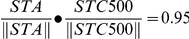
).

A similar pattern was seen when the STA was projected out of the STE prior to analysis. While the STC-NC very closely aligns with the STA for ON or OFF cells (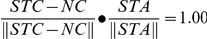, Fig. 2A), the STC-NC again clocked toward the high variance PC to better capture the structure for ON-OFF cells (Fig. 2B). However, the STC-NC did not as closely match the STC1 axis as it did in Fig. 2D. The separation observed between the STC-NC and the STC1 axes in Fig. 2B demonstrates that projecting out the STA can significantly interfere with the discovery of the direction of highest variance.

Taken together, our data demonstrate that the STC-NC can be used alone to consistently and reliably identify the stimulus feature most relevant to the classification of the cell as ON, OFF, or ON-OFF. By contrast, with conventional STC, the most important stimulus dimension is captured by different directions in the analysis, depending upon the character of the cell and whether or not the STA is projected out of the STE (Fig. 2A–D). This property complicates STC analysis, and requires careful inspection of the STE along the several dimensions defined by the STA, the low, and the high variance STC PCs in order to identify cell-type. In addition, the STC-NC linear filter strongly aligns with the largest or smallest eigenvecter of the STC analysis (Fig. 2C–D), thus the predictive capabilities of the STC-NC analysis are essentially the same as the predictive capabilities of a single-filter STC analysis. By contrast, the conventional STC analysis allows for a larger number of filters, and, obviously, with extra parameters to describe the data, it can generally be *expected* to provide better predictive capabilities. However, the benefit obtained by utilizing a larger number of STC filters is highly dependent upon the cell's RF properties. When ON and OFF subfields of the ON-OFF center cells have similar locations and temporal properties, the advantage of extra filters is small. Moreover, each additional filter places an additional load on the amount of data needed to characterize it and adds to the complexity of the analysis. Overall, the simplicity offered by the STC-NC analysis constitutes an important advantage for the STC-NC over conventional STC analysis (Table S1 compares the different methods).

Because the STC-NC identifies the single direction of greatest deviation from mean luminance in the STE, the underlying filter strength is high, and the technique is not overly data-hungry. We computed the STC-NC at 100-spike intervals for a subset of cells to study convergence using a 500-dimensional stimulus (Fig. S1). We considered an STC-NC vector to be fully converged only if the second half of the unit's spikes caused the projection of the estimated vector onto the final vector to change by <5%. In these converged cells, for 90% of the length of the estimated STC-NC vector to project onto the final STC-NC, 2600±300 spikes were needed, and for 80% of the length to project onto the final STC-NC vector, only 1400±150 spikes were required (n = 10, Fig. S1).

We further show that the LN model generated with the STC-NC more accurately reflects the underlying mechanisms of the neuron than the model generated with the STA (Fig. 2E–F). We reconstructed the LN models for the stimulus features that are represented by the directions in the high-dimensional stimulus space of panel B (Fig. 2E–F). The RF was described by the linear component of the model (Fig. 2E–F, top). The STC-NC consistently revealed a more structured spatial RF for ON-OFF cells than the STA did (Fig. 2E–F, top). Although the RFs of both the STA and the STC-NC frequently overlapped in space, their forms were different: the STA often demonstrated an unstructured bipolar RF (Fig. 2E, top) while the STC-NC demonstrated a well defined unipolar RF (Fig. 2F, top). Next, using the projection of the STE onto each linear filter (Fig. 2E–F, middle), the corresponding static nonlinearities were recovered (Fig. 2E–F, bottom). Only the STC-NC revealed a symmetric static nonlinearity reflective of the ON-OFF character of the cell (Fig. 2F).

In the LN model presented above, the STC-NC used a single filter to describe an ON-OFF center cell, with the implicit assumption that the converging ON and OFF RF centers were spatiotemporally overlapping and inverted. Gollisch and Meister (2008) demonstrated a technique by which the differing ON and OFF linear filters can be identified by computing the STA for a single cell's separated ON and OFF responses (STA-ON and STA-OFF; Fig. 2G–H). Utilizing this technique in conjunction with STC-NC analysis, which directly separated ON-type and OFF-type spikes, we observed that the ON-OFF cells possessed well-overlapped ON and OFF spatial RF components (Fig. 2G–H; insets). But while some ON-OFF cells possessed similar STA-ON and STA-OFF temporal filters (Fig. 2G), others possessed slower STA-OFF filters (Fig. 2H). Interestingly, the temporal mismatch between the ON and OFF RF components often created a triphasic STA (data not shown) [17]. Thus, while the STC-NC accurately separates the ON and OFF spikes from an ON-OFF cell, the LN model generated with this technique does not always fully describe the dynamics of the component ON or OFF pathways.

### ON, OFF, and ON-OFF classification using the STC-NC

**Figure 3:**
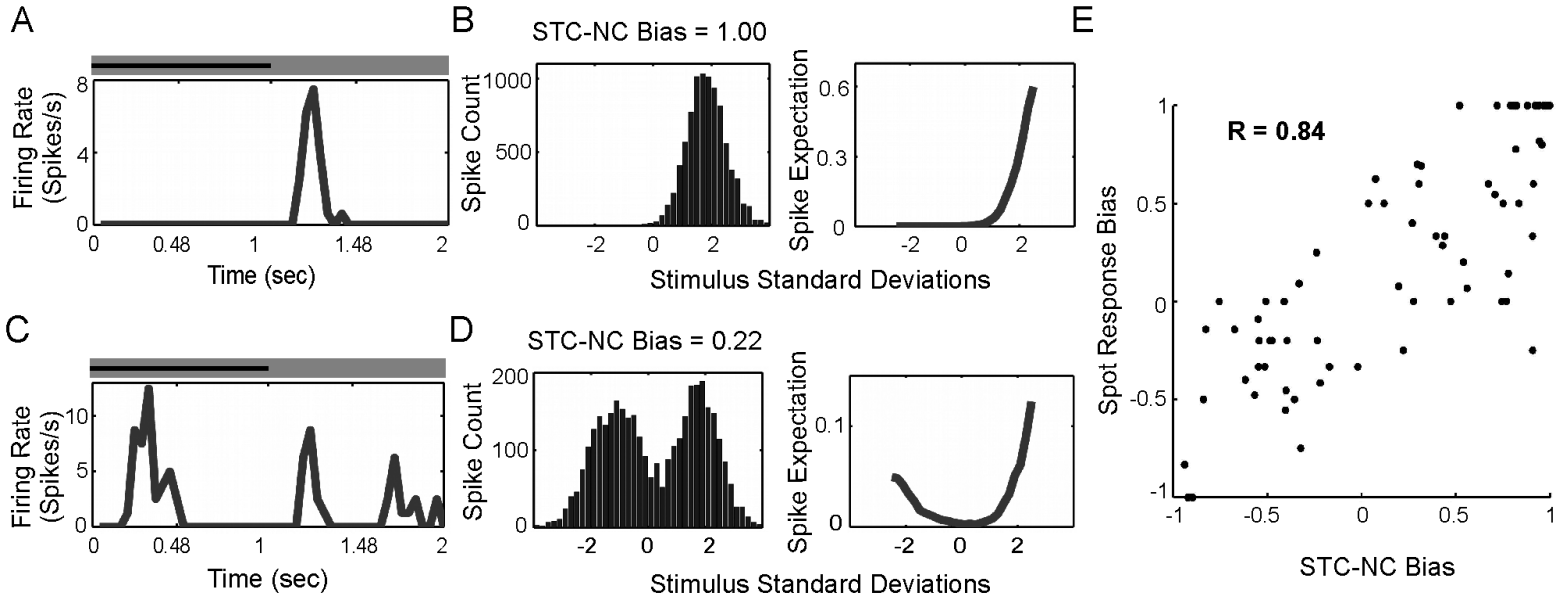
STC-NC Bias correlates well with the Spot Response Bias. (A) ON-center RGC response to spot stimulation. Time-course of stimulus above the histogram. The dark horizontal line indicates a dark spot stimulus. The cell spikes at the offset of the dark spot. (B) ON-center RGC 1D STE projection onto the STC-NC direction and resulting static nonlinearity. (C) ON-OFF RGC response to spot stimulation. The cell spikes following both the onset and offset of the dark spot. This cell also gave a delayed ON response to the offset of the dark spot. (D) ON-OFF RGC 1D STE projection onto the STC-NC direction and the resulting static nonlinearity. (E) Scatter plot of the Spot Response Bias against the STC-NC Bias showed that they were well correlated (R = 0.84).

The STC-NC Bias (see Materials and Methods) correlated well with the Spot Response Bias (Fig. 3). ON cells identified by the Spot Response Bias (Fig. 3A) had unimodal 1D STE projections onto the STC-NC, and these distributions were shifted toward positive outputs from the linear filter, creating an asymmetric static nonlinearity and a positive STC-NC Bias (Fig. 3B). ON-OFF cells identified by the Spot Response Bias (Fig. 3C) generally possessed bimodal 1D STE projections creating a more symmetric static nonlinearity (Fig. 3D) and an STC-NC Bias closer to zero. STC-NC Bias values corresponded well with Spot Response Bias values (R = 0.84, p<0.001, Fig. 3E), indicating that STC-NC analysis and responses to spot stimuli generally predict the same kind of RF center behavior.

**Figure 4:**
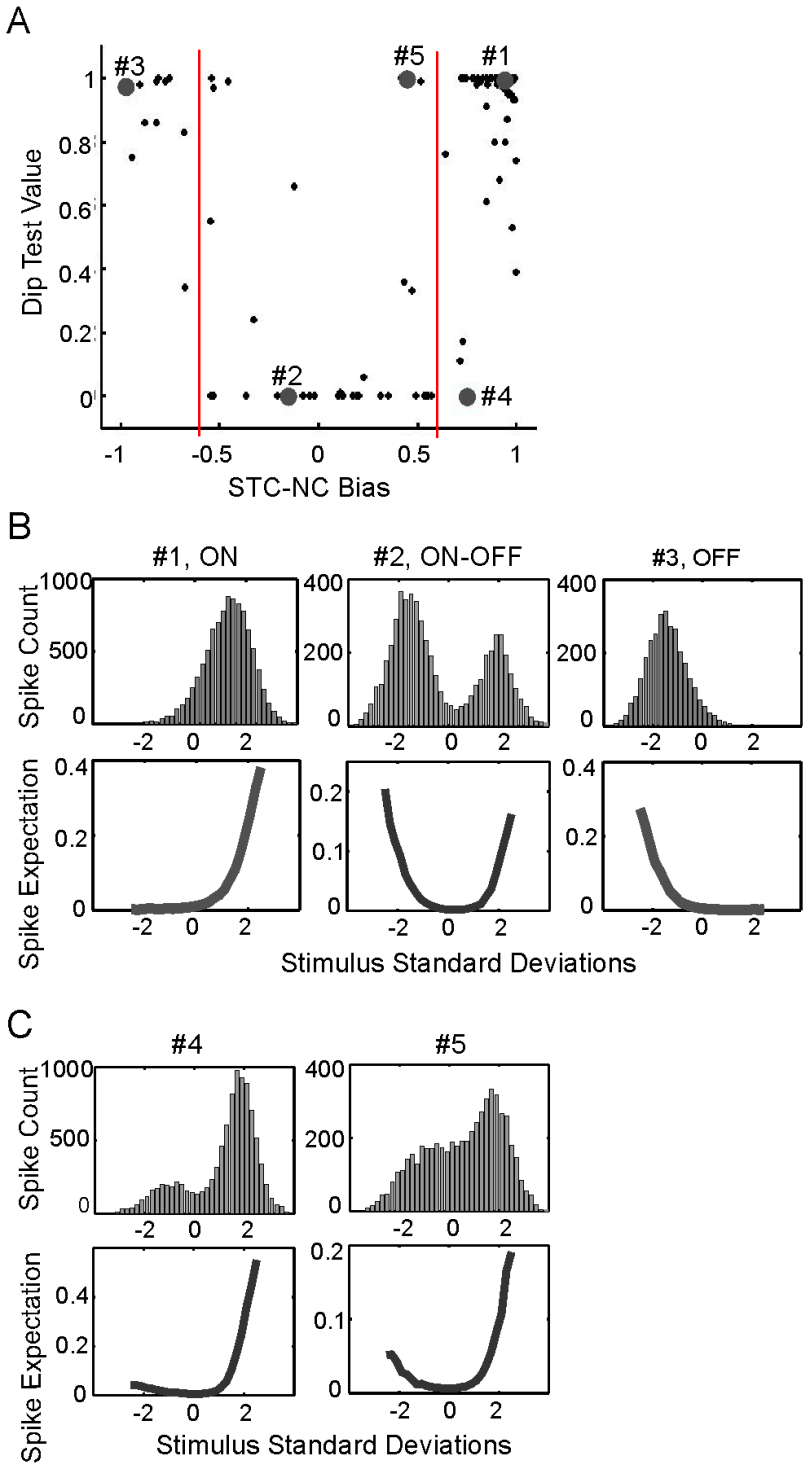
Spiking in response to both positive and negative contrasts (ON-OFF behavior) produces bimodal distributions and symmetric nonlinearities, while ON or OFF cells produce unimodal distributions and asymmetric nonlinearities. (A) Scatter plot of the STC-NC bias against the p value for the null hypothesis of a unimodal distribution. Bimodality was measured using the Hartigan and Hartigan Dip Test. Red lines at −0.6 and 0.6 show the cutoffs for ON and OFF classification. (B–C) The majority of ON and OFF cells were unimodal (e.g., B#1 and B#3) with highly asymmetric static nonlinearities, however, a few demonstrated highly unbalanced bimodality (C#4). Most ON-OFF cells were bimodal with a symmetric static nonlinearity (B#2), though some possessed less symmetric nonlinearities and were not bimodal (C#5).

To further test our classification of cells into ON, OFF, or ON-OFF subtypes using STC-NC, we examined the STC-NC Bias against a measure of bimodality. Using the Hartigan and Hartigan Dip Test, a p value was obtained for the null hypothesis that the 1D STE comes from a unimodal distribution (Fig. 4A). As expected from initial observations, cells formed three distinct clusters. OFF cells were unimodal with large negative STC-NC bias (Fig. 4B#1), ON cells were unimodal with large positive STC-NC bias (Fig. 4B#3), and ON-OFF cells were bimodal with STC-NC bias closer to zero (Fig. 4B#2). The strongly bimodal character of the ON-OFF cells is reassuring, as it indicates the presence of easily separable ON and OFF RF components.

We chose to define cells with an STC-NC Bias <−0.6 as OFF cells, cells with an STC-NC Bias >0.6 as ON cells, and those with intermediate values as ON-OFF cells. While most cells fitted nicely into these three classes, we noted that a small number of cells did not. Most cells with bimodal 1D STE distributions had a relatively symmetric static nonlinearity (Fig. 4B#2), but some were unbalanced (Fig. 4C#4). These cells possessed an STC-NC bias far from zero and were presumably not ON-OFF centered. Additionally, while the majority of cells with STC-NC bias near zero had bimodal 1D STE distributions, a few cells were determined to have unimodal distributions (Fig. 4C#5). The 1D STE distributions of many of these cells departed substantially from the normal distribution, suggesting mutimodality, but no clear secondary modes were evident. The 1D STE projections of these cells resembled the projections of previously described “ring cells” [19], suggesting that they may be sensitive to additional stimulus directions in the M dimensional stimulus space. These exceptions were comparatively rare in our cell sample and have minimal impact on the classification of cell-types in the data that follow.

### NT-3 overexpression accelerates the physiological development of RGCs after eye opening

**Figure 5:**
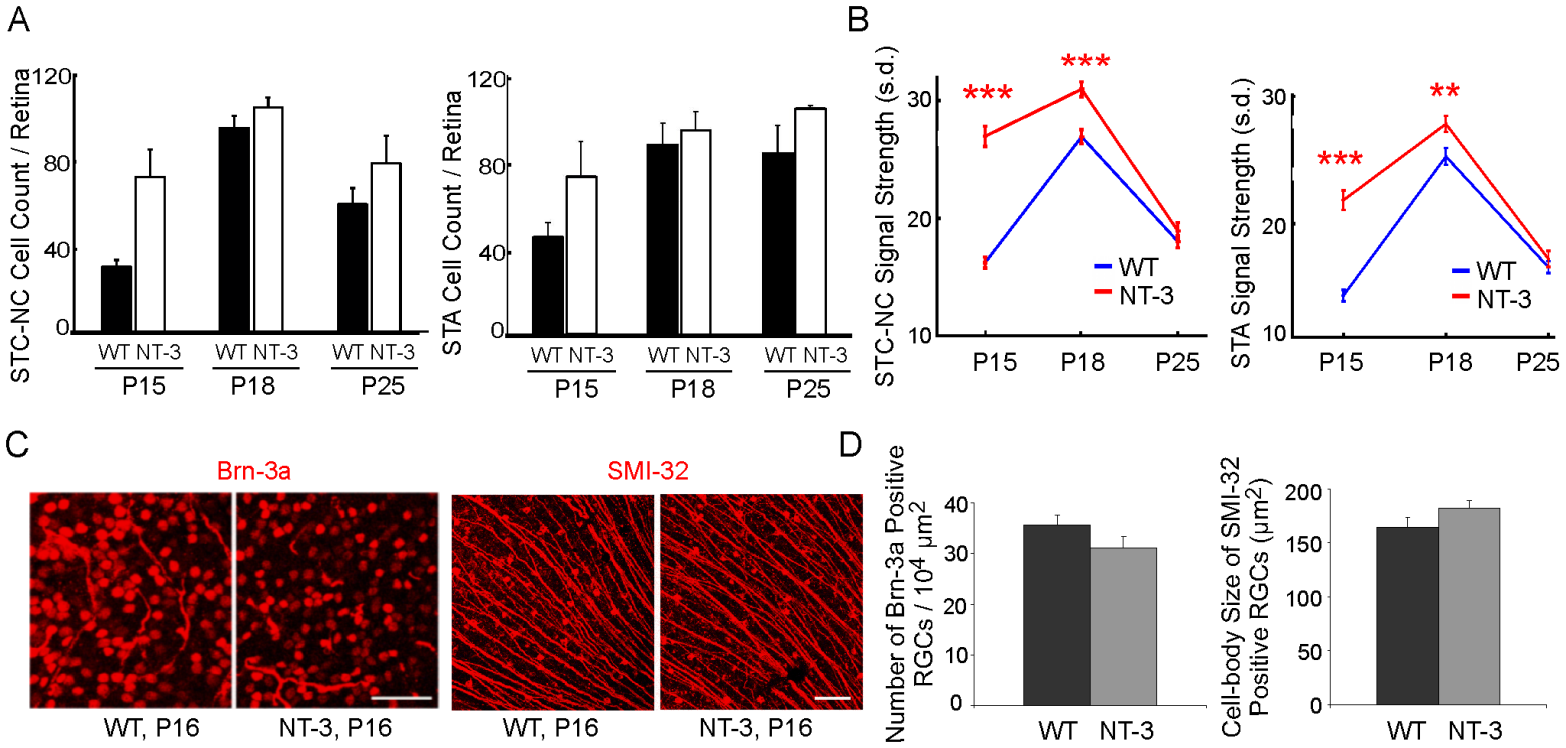
NT-3 OE mice exhibit stronger STC-NC signal strength at P15 and at P18. (A) Average cell count for NT-3 OE and WT controls at the three ages analyzed by the STC-NC or the STA method. (B) Plot of the STC-NC or the STA signal strength in s.d. against mouse age for WT and NT-3 OE mice. (C–D) Immunostainings with Brn-3a and SMI-32 antibodies demonstrated that WT and NT-3 OE retinas did not differ in total RGC number or cell body size at P16. Error bars represent S.E.M. throughout. ***: p<0.001; **: p<0.01; *: p<0.05 in Student's *t*-test.

Using the newly-developed STC-NC method, we characterized development of RF properties of RGCs in wildtype (WT) and NT-3 overexpression mice. WT retinas had poor light responses immediately following eye opening (Fig. 5A–B). At P15, fewer cells had above-threshold STC-NCs (26 cells/retina, n = 6) than at P18 (81 cells/retina, n = 4; p = 0.001 in Student's *t*-test; Fig. 5A). The number of cells per retina responding to the visual stimulus at P18 was not significantly different from P25 (51 cells/retina, n = 4; p = 0.11 in Student's *t*-test; Fig. 5A). In addition, we also noted that a large percentage of spike trains recorded at P15 lacked a defined RF and these cells were unclassifiable (Fig. S2A). About 35% of spike trains were discarded for this reason at P15, significantly higher than those at P18 (P = 0.04 in Student's t-test, Fig. S2A). When the strength of an RGC's visual response was quantified by normalizing the STC-NC's greatest absolute contrast with the standard deviation of its surrounding spatiotemporal elements, we found that the population-averaged STC-NC signal strength was greatest at P18 (p<0.0001 in One-way ANOVA test, Fig. 5B), indicating a decline in responsiveness from P18 to P25.

As demonstrated earlier, the STA analysis does not identify the RF properties of ON-OFF RGCs properly. However, as the STA represented the existing state of the art, we examined the cell number and signal strength using the STA analysis to provide a baseline against which to compare the results obtained using STC-NC analysis. Similarly, fewer cells had above-threshold STAs at P15 (45 cells/retina, n = 6) than at P18 (88 cells/retina, n = 4) and at P25 (84 cells/retina, n = 4; p<0.01 in Student's *t*-test; Fig. 5A) and the population-averaged STA signal strength was greatest at P18 (p<0.001 in One-way ANOVA test, Fig. 5B).

In NT-3 overexpression (OE) mice, cells exhibit more mature light-response characteristics than WT controls at P15. First, there were more responsive cells in NT-3 OE retinas (62 cells/retina, n = 4) than WT at P15 (26 cells/retina, n = 6; Fig. 5A), though it did not reach statistical significance with our sample size (p = 0.1 in Student's *t*-test). By P18 and P25, the number of responsive cells in NT-3 OE mice was not significantly different from WT controls (p = 0.5, Fig. 5A). Secondly, the population-averaged STC-NC signal strength was higher in NT-3 OE mice than in WT at P15 (p<0.0001) and at P18 (p<0.0001, Fig. 5B). The difference disappeared by P25 (p = 0.5, Fig. 5B). Similar results were obtained from the STA analysis that NT-3 OE mice tended to have more responsive cells at P15 (45 cells/retina, n = 6, p = 0.1) and stronger signal at both P15 (p<0.001) and P18 (p<0.01, Fig. 5B).

We confirmed that NT-3 OE mice had normal RGC morphology at P16 compared to WT controls (Fig. 5C–D). RGC were visualized by staining with Brn-3a, a transcription factor for subtype RGCs, and SMI-32, a marker for neurofilaments in subtype RGCs [9], in whole-mounted retinas at P16 (Fig. 5C). Brn-3a immuno-labels the nuclei of subtype RGCs, and SMI-32 immuno-labels the ganglion cell bodies (Fig. 5C). Overexpression of NT-3 did not affect cell density of Brn-3a positive RGCs (WT: n = 6 retinas; NT-3 OE: n = 4; P = 0.14 in Student's *t*-test), nor did it alter the cell body size of SMI-32 positive RGCs (WT: n = 4; NT-3 OE: n = 3; P = 0.18, Fig. 5D). Together, these data strongly indicate that overexpression of NT-3 accelerates the development of RGC light responses to focal stimuli after eye opening.

### Developmental decrease of ON-OFF RGC population

**Figure 6:**
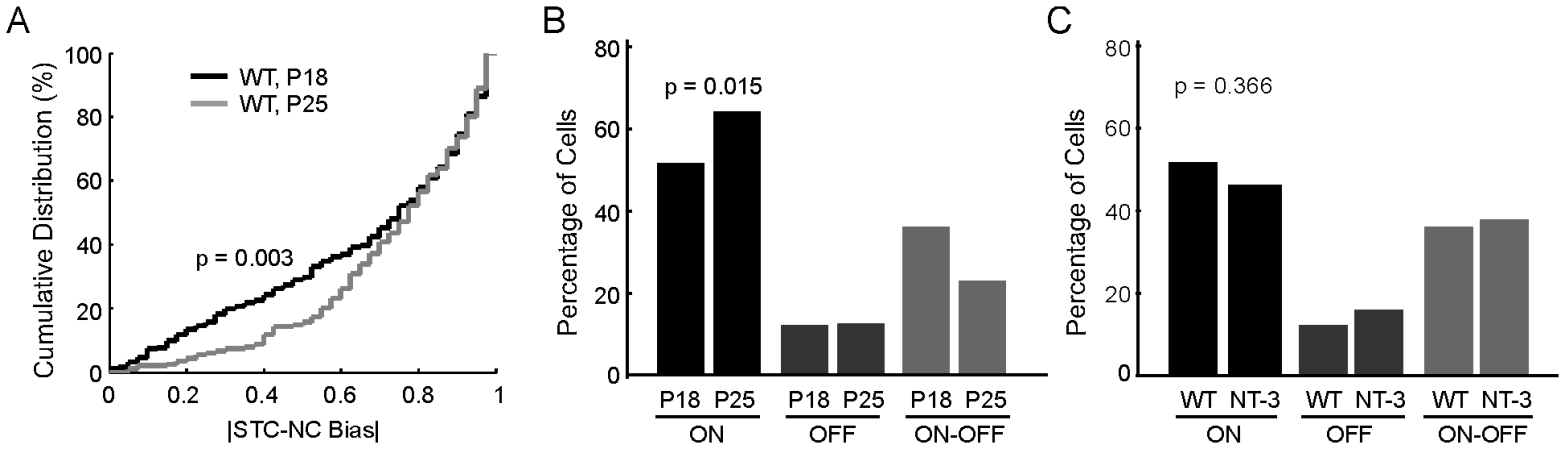
The number of ON-OFF cells decreases with age. (A) Cumulative distribution of the absolute value of the STC-NC bias for WT P18 and P25. A lower percentage of cells with ON-OFF character are observed in P25 retina (p = 0.003 in K-S Test). (B) The percentages of ON, OFF, and ON-OFF RGCs classified based on their STC-NC Bias in WT retinas at P18 and P25. The distributions differed significantly (two sample χ^2^ p = 0.015), with P25 retina possessing fewer ON-OFF cells and a corresponding increase in ON cells. (C) No difference was observed in the percentage of cell types between WT and NT-3 OE retinas at P18 (two sample χ^2^ p = 0.366).

We next examined the developmental change of the ON-OFF RGC population, which can now be accurately identified by the new STC-NC method. Previous work showed that cells with bi-stratified dendritic trees (presumptive ON-OFF cells) were gradually converted into cells with mono-stratified dendritic trees (presumptive ON or OFF cells) after eye opening [8], . We therefore quantified changes in the percentage of different RGC subtypes in the developing WT retina using STC-NC analysis, to examine whether the RGC dendritic laminar refinement correlates with the development of the RF-center properties of different RGC subtypes. Because WT retinas at P15 yielded a smaller number of recorded cells per retina (Fig. 5A), possessed a larger percentage of unmappable, visually unresponsive cells (Fig. S2A), and demonstrated lower average STC-NC and STA signal response strengths (Fig. 5A), we focused the STC-NC classification analysis on data collected from P18 and P25 mice. Cumulative histograms of the absolute value of the STC-NC Bias demonstrated that during development, the percentage of cells with strong ON-OFF character was reduced at P25 compared to P18 (p = 0.003 in K-S Test, Fig. 6A). In addition, we found a significant difference in the RGC subtype composition of WT retinas at P18 and P25 (Fig. 6B, two sample χ^2^ p = 0.015). At P25, the percentage of ON-OFF cells was reduced compared to P18, and the percentage of ON cells was increased correspondingly (Fig. 6B). The percentage of OFF cells was similar for the two ages (Fig. 6B). Our data demonstrate therefore that the percentage of ON-OFF center RGCs decreases while the percentage of ON center RGCs increases with age after eye opening.

We compared WT to NT-3 OE retinas at P18 and found no significant changes in the percentage of the three RGC subtypes in NT-3 OE retinas (Fig. 6C, two sample χ^2^ p = 0.366). Prior work has shown that NT-3 OE mice had fewer cells with bi-laminated dendritic trees at P13 compared to age-matched WTs, however, this difference disappeared by P28 [9]. In this study, we also found, consistent with the anatomical results, that the distribution of the three physiological RGC subtypes was the same in NT-3 OE retinas and WT controls at P25 (data not shown).

### Maturation of RF center size in WT retina

**Figure 7:**
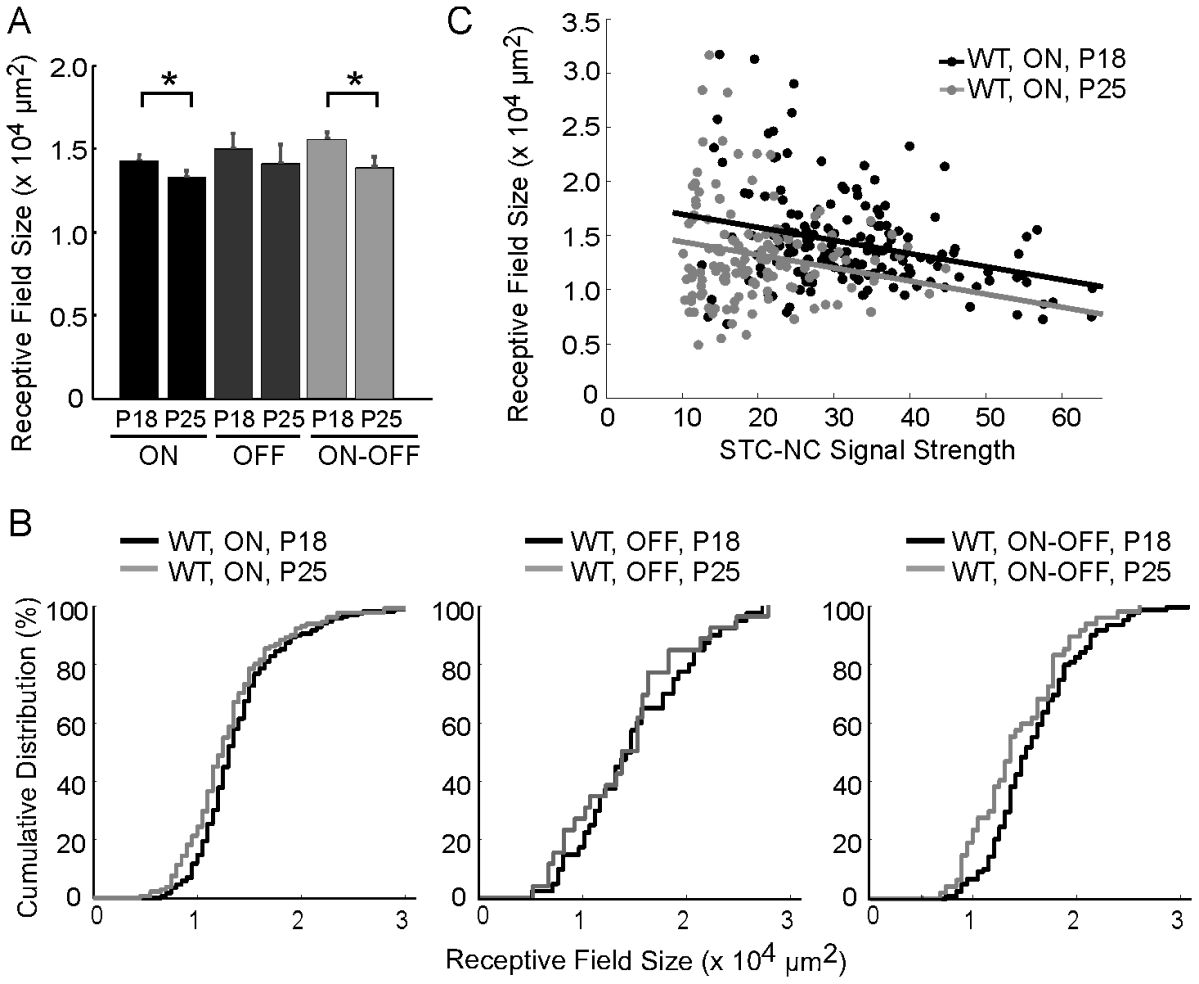
STC-NC analysis measures a reduction in the RF center size for both ON and ON-OFF cells during WT development. (A) Both ON and ON-OFF RGCs show a decrease in RF center size at P25 compared to P18, while the size of OFF RGC RF centers is unchanged. (B) Cumulative distributions of RF center sizes for ON, OFF, and ON-OFF cells in WT. *: p<0.05; ***: p<0.001 in Wilcoxon rank sum test (same in Fig. 8). (C) The correlation between the STC-NC signal strength and RF size is negative for WT P18 (black) and P25 (gray) ON cells. The ANCOVA technique removes the effect of the confounding STC-NC signal strength prior to calculating the significance of differences in RF size due to grouping. The parallel lines represent best fits. Following standard ANCOVA analysis, the parallel condition was enforced after demonstrating that the slopes of the lines of best fit through the two data sets were not significantly different and that P25 had smaller RF sizes than P18 for WT ON cells ( p = 4×10^−5^ in ANCOVA test).

With the superior cell classification and improved RF mapping provided by the STC-NC, we examined the development of RF center sizes in WT retinas in a subtype-specific manner (Fig. 7). We found that ON-OFF cells exhibited a significant decrease in RF center size at P25 compared to P18 (Fig. 7B). The mean RF center size for ON-OFF cells at P25 was 13.9±0.6×10^3^ µm^2^, significantly lower than that at P18 (15.6±0.4×10^3^ µm^2^, p = 0.03 in Wilcoxon rank sum; same below). The RF center sizes of ON cells also decreased from P18 to P25 (P18: 14.3±0.3×10^3^ µm^2^; P25: 13.3±0.4×10^3^ µm^2^; p = 0.03; Fig. 7B). OFF cells displayed a slight trend towards smaller RF center size too, but this change was not statistically significant (P18: 15.0±0.8×10^3^ µm^2^; P25: 14.1±1.0×10^3^ µm^2^; p = 0.64, Fig. 7B). These data demonstrate that the center size of ON and ON-OFF cells decrease with age, while the size of OFF-cell centers is unchanged.

To rule out the possibility that a smaller RF center was due to a weaker center response, we plotted the STC-NC signal strength against RF size for WT ON cells at ages of P18 and P25 (Fig. 7C). We found that the RF sizes demonstrated weak but negative correlations with their signal strength (P18: R = −0.34, P≤10^−4^; P25: P25: R = −0.11, P = 0.23; Fig. 7C). Our data suggest that increased signal strength is associated with smaller, but not larger, RF size. To isolate the significance of the group variable alone, we thus utilized the Analysis of Covariance technique (ANCOVA) to compensate for variations in STC-NC signal strength. We still found that P25 ON cells and ON-OFF cells were significantly smaller (ON: p = 4×10^−5^; ON-OFF: p = 5×10^−3^), and that OFF cells were unchanged (p = 0.68 in ANCOVA test).

For P15 WT animals, we were able to characterize, on average, only 26 cells per retina with STC-NC analysis. Dividing this number among the three classes, we might expect to record roughly 13 ON cells, 10 ON-OFF cells, and only 3 OFF cells (Fig. S2B). Nevertheless, we were able to collect data from enough samples to analyze their RF center sizes (Fig. S2C–F). We found that the RF sizes for OFF and ON-OFF cells increased from P15 to P18 (OFF: n = 23 cells, p = 0.04; ON-OFF: n = 41 cells, p = 0.003 in Wilcoxon rank sum, Fig. S2C–F). For ON cells, the change was not significant (n = 91 cells, p = 0.09, Fig. S2D). Taken together, our data suggest that different subtypes of RGCs exhibit different growth patterns after eye opening and cannot be therefore passive processes resulting simply from growth of the eye.

We also confirmed that with STC-NC a single bivariate Gaussian function fitted the RF center better that it did for the STA when considered over all cell-types (Fig. S3). For ON and OFF cells, both the STA and the STC-NC accurately described the spatial RF structure, and as expected, the STA and the STC-NC gave similar RF center sizes for these RGC subtypes (Fig. S3). For ON-OFF cells, however, the STA failed to characterize the RF center (Fig. 2B, E); the unstructured center given by the STA did not fit a bivariate Gaussian well and the resulting estimate of center size was poor. The STA measurement of RF center size for ON-OFF cells was significantly smaller than the STC-NC measurement (p<0.001, Fig. S3). The STA therefore poorly estimates ON-OFF cell RF center size — a problem remedied by the use of STC-NC analysis.

### Reduced RF center size of ON-OFF cells in NT-3 OE mice

**Figure 8:**
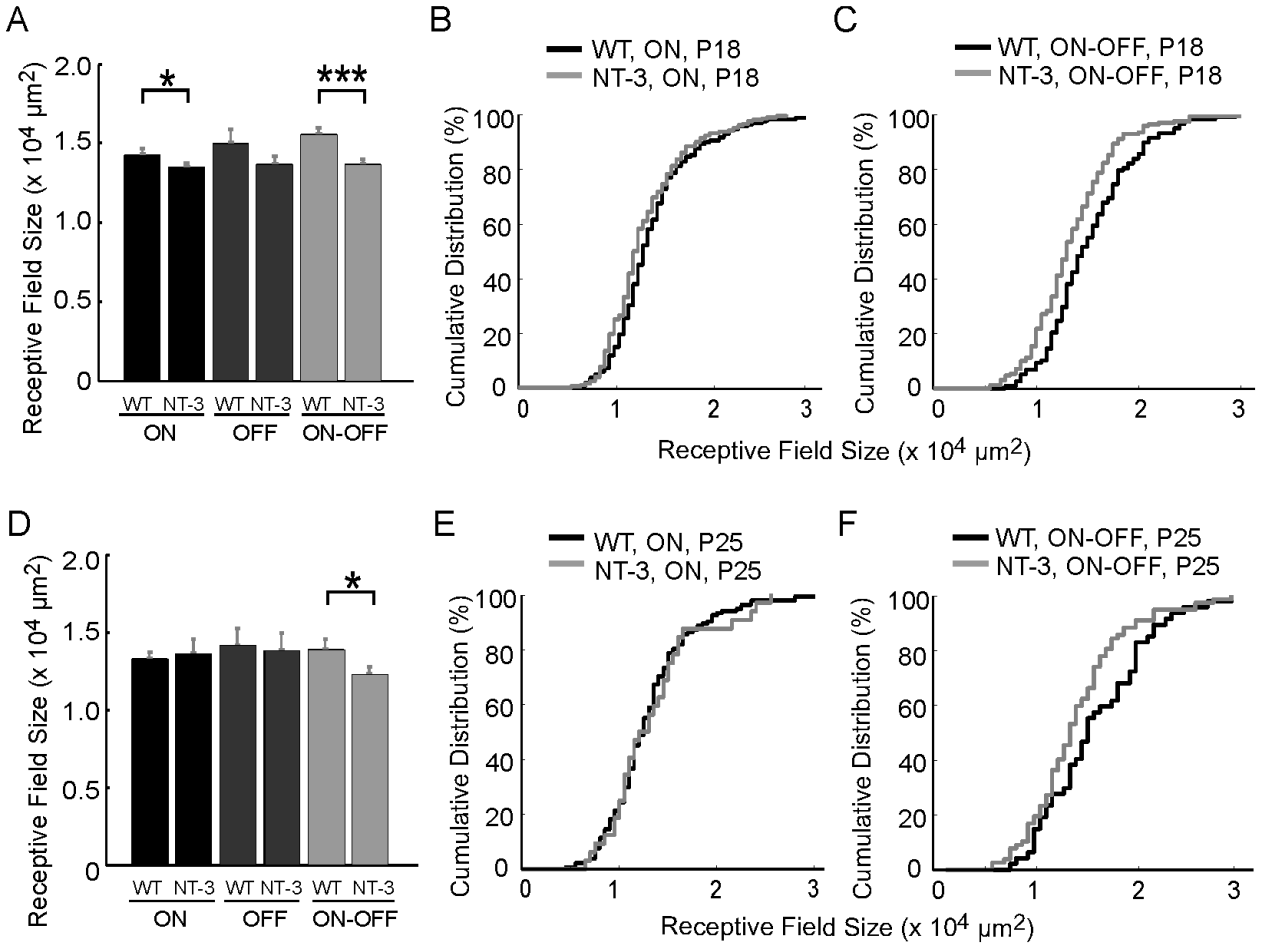
STC-NC analysis reveals smaller RF center size of ON-OFF cells in NT-3 OE mice. (A) ON and ON-OFF RGCs in NT-3 OE retinas had smaller RF centers at P18 than WT mice. (D) At P25, only ON-OFF RGCs in NT-3 OE had smaller RF centers compared to WT. (B–C, E–F) Cumulative distributions of RF sizes for ON (B, E) and ON-OFF cells (C, F) in WT and NT-3 OE retinas at P18 (B–C) and P25 (E–F).

We next investigated whether NT-3 regulates the RF center size of different RGC subtypes after eye opening (Fig. 8). With the STC-NC, we found that ON-OFF cells in NT-3 OE mice exhibited significantly smaller RF centers compared to WT retinas at P18 (WT: 15.6±0.4×10^3^ µm^2^; n = 118; NT-3 OE: 13.7±0.3×10^3^ µm^2^, n = 170; p<0.001 in Wilcoxon rank sum, Fig. 8B–C). ON cells also possessed smaller center sizes in NT-3 OE mice at P18, although this difference was less pronounced than it was for ON-OFF cells (WT: 14.3±0.3×103 µm^2^; n = 169; NT-3 OE: 13.5±0.3×103 µm^2^, n = 208; p = 0.04; Fig. 8A–B). Although the RF center sizes of OFF cells tended to be smaller in NT-3 OE mice compared to WT the difference did not reach statistical significance with our sample size (WT: 15.0±0.9×103 µm^2^; n = 40; NT-3 OE: 13.7±0.5×103 µm^2^, n = 72; p = 0.24, Fig. 8A–B).

At P25, ON-OFF cells continue to have significantly smaller RF centers in NT-3 OE mice (Fig. 8E–F). The mean center size of ON-OFF cells at P25 in NT-3 OE mice was 12.3±0.5×10^3^ µm^2^, significantly smaller than that of WT controls (13.9±0.6×10^3^ µm^2^, p = 0.04 in Wilcoxon rank sum, Fig. 8E–F). The small difference between WT and NT-3 OE mice in the center size of ON cells at P18 had disappeared by P25, and what difference in center size there might have been for OFF cells at P18 was also absent by P25 (ON: p = 0.83; OFF: p = 0.92, Fig. 8E). With ANCOVA statistical test to compensate for the increases in STC-NC signal strength in NT-3 OE mice, we continued to find that at P18 NT-3 OE retinas had smaller ON-OFF cells than WT controls (p = 0.03), but not for ON and OFF cells (ON: p = 0.28; OFF: p = 0.19). This same pattern was observed at P25, where ON-OFF cells in NT-3 OE retinas were again smaller than WT controls (ON-OFF: p = 0.04; ON: p = 0.42; OFF: p = 0.85 in ANCOVA test). Previous work has shown that bistratified ON-OFF cells have smaller dendritic field sizes in NT-3 OE mice [9]. With the STC-NC analysis, we have shown here that overexpression of NT-3 accelerates the developmental decrease of RF center size in ON-OFF cells, consistent with the earlier anatomical work.

## Discussion

### Advantages and limitations of STC-NC

RGCs are often modeled with a linear filter followed by a static nonlinearity (the LN model). The visual stimulus is convolved with the linear filter and the output passed through a static nonlinearity which controls the expectation of spike discharge [15], [16], [19], [21]. The STA is often used as the linear filter in LN models of ON and OFF center RGCs [16], [18]. Because the STA is the average stimulus preceding a spike, if the underlying nonlinear mechanism of an LN neuron is highly symmetric, as one might expect for ON-OFF cells, the STA is unable to recover the linear filter [21]. STC analysis is a technique used in combination with the STA to identify additional linear filters for neurons in the visual system [19]–[21]. A full STC analysis can be particularly powerful when used to uncover multiple filters from the complex RFs of neurons in the visual cortex [20]. When applied to retinal neurons, STC is also capable of identifying the filters used by ON-OFF RGCs (Fig. 2). But, when applied to the entire population of RGCs, the primary RF mechanism is not captured by a single STC filter. Instead, it may be described by any one (or combination) of the STA, the low, or the high variance eigenvectors, depending on the character of the RGC and whether or not the STC analysis is performed in a space perpendicular to the STA (Fig. 2).

In this study, we demonstrated that the STC-NC greatly simplifies classification of RGCs and that it has an intuitive interpretation. Performing PCA on a non-centered moment matrix is justified, particularly when the zero vector (mean luminance) is an important reference [24], [27]. Because this technique is not centered with the STA, it maximizes the second moment of the STE about mean luminance, not the variance (which by definition is the second moment of the STE about its own mean). Using a non-centered second moment matrix allows us to find the single direction of maximal deviation from mean luminance regardless of whether that deviation is asymmetric (ON or OFF) or symmetric (ON-OFF). However, the STC-NC is aimed primarily at high throughput cell classification, not spike prediction, where multiple-filter models (including full STC analysis) will provide greater accuracy [17], [19]. For example, a single filter STC-NC LN model cannot describe the differing temporal dynamics sometimes observed in the separate ON and OFF RF filters of an ON-OFF cell (Fig. 2G–H). But even with the limitation of a single filter to model ON- and OFF-type spikes, the STC-NC may form the foundation for more sophisticated, multi-filter or multi-step analyses.

In addition to our standard 100 µm×100 µm checkers, we have also used 60 µm×60 µm checkers, which potentially provide better resolution in mapping the spatial RF (Fig. S4). The cells in Fig. S4A–B were exposed to both 60×60 µm and 100×100 µm checker stimuli for equal durations of time. As expected, the RF maps showed improved resolution with smaller checker sizes. However, as the checker size decreases, the response strength was also decreased with reduced spike counts and lower spike expectations (Fig. S4A–B). Moreover, for a large number of cells, we were only able to map the RF with the 100×100 µm checkers because the smaller checkers did not elicit a strong enough response. For example, at P25, we collected a total of 204 WT cells with RFs mapped by 100×100 µm checkers, but only 70 RFs were mapped with the 60×60 µm stimulus. Despite these limitations, we observed a similar development trend from P18 to P25 upon comparing the 60×60 µm and 100×100 µm checker data (Fig. S4C). In WT, the RF sizes of ON and ON-OFF cells were decreased from P18 to P25 (ON: p = 1.41×10^−4^; ON-OFF: p = 3.25×10^−4^), but the size of OFF cells remains unchanged (p = 0.59 in Wilcoxon rank sum test, Fig. S4C).

We compared RF center measurements made by the STC-NC with those made by the STA (e.g. [18]). Kerschensteiner and his collegues (2008) found that the RF 1σ radii for ON-center and OFF-center cells ranged from 60–120 µm in 2–3-month old mice using 66 µm×66 µm checkerboard stimuli. Here, we showed that at P18, our data correspond to 1σ radii for ON-center and OFF-center RF centers of 70 µm and 67 µm, respectively (Figs. 6–7), which fall within the range of the published measurements [18]. Moreover, we demonstrate here that the STA provides inaccurate estimates of ON-OFF RF center sizes (Figs 1–2 and Fig. S3) and that measurements based on the STC-NC are more accurate. The STC-NC better describes the ON-OFF character of the RF center, correlating well with the results of spot stimulation (Fig. 3E), it reveals a well-structured spatial RF center (Fig. 2F), and is capable of classifying cells efficiently into ON, OFF, and ON-OFF types (Fig. 4A).

### Does RGC dendritic structural refinement correlate with functional maturation?

The correlation between the RGC dendritic structure and its RF properties is not straightforward. Based on RGC dendritic morphology about 10–14 subtypes of RGCs have been identified in mouse [28]–[30]. Classification of RGCs based on physiological properties is incomplete, as is the correspondence between morphological and physiological types [1], [2]. For some subtypes of RGCs a match between dendritic and RF properties can be made [31]–[34]. For example, one class of OFF RGCs has asymmetric dendritic arbors aligned in a dorsal-to-ventral direction across the mouse retina that matches their responses to visual stimuli moving in a soma-to-dendrite direction [32]. On the other hand, studies in the rabbit retina have shown that about 10% of the direction-selective cells have RFs displaced toward the preferred direction, while their dendritic structures exhibit no obvious corresponding relationship [35].

During postnatal development, RGC dendritic laminar refinement somewhat correlates with the functional separation of ON and OFF pathways. Using full-field flash stimulus, Tian and Copenhagen showed that ON-OFF cells decrease from 76% before eye-opening to 40% immediately after eye opening to 22% at P28. In this study, we provide the reliable identification of the ON-OFF subtype and show that cells with ON-OFF centers decrease from 35% of the RGC population at P18 to 24% at P25 (Fig. 6B). Based on RGC dendritic morphology, Landi et al. (2007) showed that 66% of RGCs were bistratified (presumed ON-OFF) at P10, and this percentage decreased to 54% at P16 and 31% at P30. These results are generally consistent with our previous studies of RGC dendritic laminar refinement where the percentage of RGCs possessing bi-laminated dendritic structure decreases from ∼50% to ∼35% from P13 to P28 [8]. In adult mice (>P27), about 50–60% RGCs are ON RGCs ([8], [11], [30], but see [28] which showed 50% of RGCs are ON-OFF RGCs). Unlike in cats, which have roughly 50–50 ON vs OFF cells [2], much fewer OFF cells (5–15%) are reported in mice [8], [11], [30]. In addition, RGC response can be also characterized along different dimensions other than ON vs. OFF, e.g. sustained vs. transient, and brisk vs. sluggish [2]. It is of great interest to characterize further these properties of RGCs by MEA with new analytical tools.

At the same time, refinement of RGC dendritic arbor does not always correlate with the functional maturation. In the developing turtle retina, intense dendritic growth occurs before RGCs became sensitive to light, and a weak correlation is found between physiological RFs and dendritic arbor structure [36]. In kitten, the arbors of gamma RGCs are similar to their adult counterpart, while the dendritic fields of alpha cells in the peripheral retina reach their adult dimensions three weeks after birth, around which time beta cells begin to expand [6], [7]. By contrast, most cells respond to light first at P10 with RF centers invariant or in some cases larger during postnatal development than in the adult [37]. In developing mouse retina, the size of the dendritic field is typically somewhat larger than the size of the RGC RF center determined by STC-NC analysis. For all RGCs, the dendritic field size at P13 (mean: 17.6±1.1×10^3^ µm^2^) is about 16% larger than the 1σ contour of their RF center at P18 (mean: 14.8±0.3×10^3^ µm^2^). For ON RGCs, the mean dendritic field size at P13 is 16.2±1.3×10^3^ µm^2^ [9], about 12% larger than the mean 1σ area of their RF center at P18 (14.3±0.3×10^3^ µm^2^). Interestingly, during the two weeks after eye opening, the dendritic field size of ON RGCs increases almost 47% (P28: 23.9±1.0×10^3^ µm^2^), while their 1σ RF center size at P25 (13.3±0.4×10^3^ µm^2^) is 7% smaller than it was at P18. These data suggest that the relationship between dendritic field extent and RF center size that seems to be quite robust in adult retina is less clear-cut during development when synaptic contacts are being established and refined.

The developmental mechanisms of RGC dendritic structural refinement and their functional maturation remain to be elucidated. The early dendritic arborization and synaptic formation of RGCs are generally thought to be regulated by intrinsic growth programs [1], [38]. For example, recent studies have shown that immunoglobulin superfamily (IgSF) adhesion molecules–Dscam, DscamL, Sidekick-1 and Sidekick-2–are expressed in distinct IPL sublaminae of chick retinae [39]. Loss- and gain-of-function studies *in vivo* showed that these IgSF members participate in determining the IPL sublaminae in which synaptic partners arborize and connect [39]. In later development, environmental signals are involved in the regulation of RGC dendritic maturation [1], [8], [38]. Time-lapse imaging experiments have revealed that RGCs take an active role in sampling the local retinal environment and in establishing functional synaptic contacts with amacrine and bipolar neurons by extending and retracting dendritic filopodia [40]. Selective removal of ON input causes a reduced rate of synapse formation rather than an increase in synapse elimination, creating ON-OFF RGCs with fewer synapses in their ON arbors without affecting OFF arbor structure [41]. Contrary to this view, other studies have suggested that the formation of synaptic connections between RGC dendrites and other neuronal processes in the IPL is established through the elimination of superfluous processes [42], [43]. For example, large field type-I rat RGCs exhibit extensive branch loss [43].

### Neurotrophic mechanisms underlying RGC development

Neurotrophins modulate dendritic development in many nervous systems [44]. We have previously shown that BDNF and NT-3 play overlapping roles in RGC dendritic laminar refinement and distinct roles in subtype-specific maturation of RGCs [8], [9]. Indeed, many studies have suggested that BDNF can exert multiple roles on RGC dendritic development through different mechanisms [38]. For example, retinal BDNF levels directly affect the complexity of RGC dendritic arbors in *Xenopus* [8], [45]. BDNF could also regulate the morphology and function of amacrine cells which in turn influence RGC dendritic connectivity indirectly [46], [47]. Because the expression and release of BDNF is regulated by visual experience, BDNF may modify RGC dendritic laminar refinement by altering synaptic connectivity between RGCs and other input neurons [8]. Finally, target-derived BDNF could retrogradely affect RGC dendritic structure and function [45], [48].

Compared to the intensive studies on BDNF, little is know about the roles of NT-3 in retinal development. In mouse retina, NT-3 is expressed both pre- and postnatally and its expression is unaffected by visual experience [9], [49]. In slice cultures of cortical neurons, NT-3 stimulated dendritic growth in layer 6 and inhibited BDNF-stimulated dendritic growth in layer 4 [50]. In developing chick retina, NT-3 regulates RGC survival and the structure of the IPL [51]. Here we have shown that NT-3 regulates RF properties of RGCs during postnatal development. Our previous work showed that dendritic trees of RGCs that bistratify in the IPL are smaller in NT-3 OE mice, while those which are monostratified are not [9]. Here we have shown that the RF centers of ON-OFF but not ON or OFF center cells are smaller in adult NT-3 OE mice (Fig. 8), consistent with the earlier anatomical work. Future studies are needed to identify whether and how the refinement of dendritic field size and RF size is translated into the maturation of visual function in WT retinas and when the neurotrophin signaling is perturbed.

In conclusion, we have established the STC-NC analysis as a method for the characterization of RGC subtype RF properties, and demonstrated that NT-3 regulates the functional development of ON-OFF center RGCs. Our study provides a basis for future examination of how neurotrophic signaling pathways modulate RGC RF properties.

## Materials and Methods

### Animals

Transgenic mice expressing NT-3 driven by the alpha A-crystallin promoter from the lens (labeled as NT-3 OE mice) on the BALB/c genetic background [52] were crossed more than ten times with C57BL/6, so that the NT-3 OE mice were mainly on a C57BL/6 background [9]. All animal procedures conformed to the guidelines in the Use of Animals in Neuroscience Research from the NIH and were in accordance with protocols approved by the Northwestern University IACUC.

### Multi-Electrode Array (MEA) recordings

NT-3 OE and WT mice were euthanized by direct cervical dislocation, and the eyes were placed into an oxygenated (95% O_2_, 5% CO_2_) artificial cerebrospinal fluid (ACSF). Under infrared illumination, a single retina was isolated and about one fifth of the retina was cut for each experiment. The retinal ganglion cell layer (GCL) was then placed into contact with a 60-channel multielectrode array (Multichannel Systems Gmbh, Fig. S5) [13]. The retina was perfused with ACSF and maintained at 33–34°C during the entire recording session. An image from a computer monitor was projected onto the retina. As the retina responded to visual patterns on the monitor, voltage signals from the microelectrodes were amplified by the MCS preamplifier (bandpass 1–5000 Hz) and recorded (MCRack software). Spike waveforms were collected using a voltage threshold and sorted with Plexon Offline Sorter to isolate spike trains [13]. Spike timestamps were then exported to Matlab, where custom analyses were applied individually to each RGC's spike train.

To characterize the spatiotemporal visual responses of the RGCs at different developmental ages, three monochrome stimuli were applied: a full-field flash, a spatiotemporal Gaussian white noise, and a flashing spot stimulus. The mean luminance for all stimuli was 2 cd·m^−2^. The full-field flash was a repetitive binary stimulus consisting of 2 seconds with the light OFF (0 cd·m^−2^) followed by 2 seconds with the light ON (4 cd·m^−2^). The white noise stimulus appeared as a flickering grayscale checkerboard pattern with random spatial and temporal structure, which was composed of 100 µm×100 µm square checkers. A new random luminance was assigned to each checker every 33 ms. The Gaussian distribution from which luminance values were randomly drawn was centered at 2 cd·m^−2^ with a standard deviation of 0.78 cd·m^−2^, causing the distribution to be truncated at ±∼2.5 standard deviations. The spot stimulus utilized the same checkerboard pattern as the white noise, however, the entire frame was assigned to mean luminance, and a single randomly chosen checker was flashed either ON (4 cd·m^−2^) or OFF (0 cd·m^−2^) every second. Both ON and OFF spots covered each location a minimum of 20 times, and the location and polarity of the flashed checker was randomized.

### Data analysis for the full field flash stimulus

Peristimulus time histograms (PSTHs) and raster plots of individual units were generated. The Response Dominance Index (RDI) was calculated from the transient peak spike rates during the first quarter of the ON (R_ON_) and OFF portions (R_OFF_) of the full field stimulus by the following equation [11], [22]:
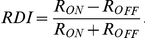



The value of the RDI ranges from −1 to 1. Cells with an RDI near 1 possess an ON-dominating response, those with an RDI near −1 possess an OFF-dominating response, and the cells with an intermediate RDI near 0 possess a more balanced ON-OFF response.

### Spike-Triggered Analysis (STA)

Spikes driven by the Gaussian white noise stimulus were placed into 33 ms bins defined by the stimulus refresh cycle. The 30 frames preceding each spike were linearized to form a vector, **s**_n_, which describes the spatiotemporal stimulus preceding the n^th^ spike in the spike train. It has length 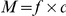, where *f* is the number of frames preceding the spike that are captured for analysis, and *c* is the number of checkers per frame. The Spike-Triggered Ensemble (STE), which is the set of spatiotemporal visual stimuli preceding all spikes in a neuron's spike train, was constructed as an NxM matrix, **S**, by collecting all **s**_n_ into the rows of **S**. The STE is a subset of the entire Raw Stimulus Set (RSS), which is the collection of all the spatiotemporal stimuli, **s**, that were presented to the retina. Each element of the vector **s** possesses a value representing the deviation from mean luminance (2 cd·m^−2^), and over all the **s** vectors in RSS, these values form a Gaussian distribution centered at zero. The STE and RSS collections exist in an M-dimensional space, which corresponds to the number of independent elements in **s**, and they often possess different means. The identification of statistical differences between the STE and the RSS in this M-dimensional space forms the basis for spike-triggered neural characterization [21].

The STA is simply the vector average of the STE.
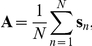
where N is the total number of spikes in the spike train [16]. Each element of the vector **A**, therefore, is the average of all the values stored in the corresponding column of **S**. The average of the RSS is the zero vector, but in general the STA differs greatly from zero, and it therefore represents a stimulus feature that evokes a spike from the neuron.

During the analysis, the standard deviation of the elements in each cell's STA was calculated. A cell was considered to have a non-responsive STA if no single element of its STA exceeded six standard deviations in magnitude. In addition, we utilized the location of the STA receptive field to identify potential duplicate recordings of single RGCs on neighboring electrode channels. Crosscorrelation plots were generated to confirm and reject duplicate spike trains. In general, ∼40% duplicates for hexagonal MEA and for rectangular MEA ∼15% duplicates of spike trains with above-threshold (“mappable”) STAs were removed.

### Spike Triggered Covariance - Non-Centered (STC-NC) analysis

STC analysis is implemented by performing a principal component analysis (PCA) on the STE. PCA can be achieved by eigendecomposition of the covariance matrix, which generates eigenvectors that are then sorted by their eigenvalues to identify directions of large and small variance. Because the STC estimation error is proportional to 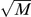 [21], we windowed the stimulus vectors, **s**_n_, around the central checker of the STA. This step reduced the spatial extent of the stimulus, but did not affect the number of frames captured. We used a 5×5 window, and the effect was to create new **s**_n_ vectors with smaller length, thereby reducing the dimensionality, M, of the stimulus. A shorter 666 ms (20 frames) time period was also used. The covariance matrix, **C**, is an M×M matrix, and it was calculated with the following equation:
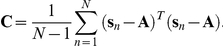

**C** is a positive definite matrix as long as 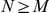, otherwise it is positive semi-definite. Subsequently, eigendecomposition was performed on **C**, and the eigenvectors were sorted according to their corresponding eigenvalues. This process allowed us to find a new basis set for the stimulus space, in which the directions were ordered according to STE variance.

Traditionally, during PCA, the covariance matrix is created by computing the outer product of the mean-centered STE with itself. Eigendecomposition is then performed on the covariance matrix, and the resulting eigenvectors represent the principal components. However, in situations where classification is the primary goal, eigendecomposition of a non-centered moment matrix can be justified when the zero vector is an important point of reference [24], [27]. We computed the non-centered second moment matrix **M**, with the following equation:
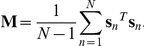



In the case of a non-centered moment matrix, the eigenvector with the greatest eigenvalue maximizes the second moment of the STE *around zero* – not the variance, which is the second moment *around the mean*. The resulting eigenvectors are often referred to as non-centered principal components [27]. It is important to note that since the covariance matrix is not used, these eigenvectors are not strictly principal components in the STC-NC technique.

Because the STC-NC is only a 5×5 window, the RF makes up a large portion of the STC-NC vector. For this reason, to get a better measure of how strong the RF center signal was compared to the surrounding noise, the maximum contrast of the STC-NC vector was divided by the standard deviation of the outermost ring of pixels in the STC-NC (not the standard deviation of the full vector). In the analysis, we excluded RGCs with low STC-NC signal which had visual responses that were weak or non-existent.

The code for the core STC-NC algorithm, along with sample data, is available at: http://code.google.com/p/non-centered-spike-triggered-covariance/

### Identification of ON, OFF, and ON-OFF character of RGCs

In a LN model for RGC stimulus-response transduction, the scalar output from one or several linear filters is used as input for a static nonlinearity, which predicts the probability that the neuron will spike. The static nonlinearity is a function that maps from k-dimensional space to a 1-dimensional space (ℜ^k^ ⇒ ℜ^1^), where k is the number of linear filters used. We primarily used a single filter in this study. Thus, the nonlinear transform was computed in a few simple steps [16], [21]. First the output of the linear filter for all **s** in RSS was calculated and binned to form a vector **L**_RSS_. For Gaussian white noise, the resulting distribution very closely approximated the Gaussian distribution used for stimulus generation. Next, the output of the linear filter for all **s**_n_ in the STE was calculated and binned to form **L**_STE_. Finally, **L**_STE_ was divided, element-by-element, by **L**_RSS_. The resulting vector, **N**, held the fractional number of spikes expected in response to a given input from the linear filter. The vector **N** deviates from predicting spike probability only in the sense that the values in this vector can exceed 1 if more than 1 spike is expected within the binned time period. Prior to this calculation, we multiplied all OFF-type linear filters by −1, forcing them to appear as ON-type filters. This trick ensures that all stimuli that represent a light offset in the RF center create a negative scalar output when convolved with the linear filter, and vice versa for stimuli representing light onset in the RF center. This was done to properly orient the polarity of the 1D STE and RSS projections onto the linear filters, which in turn orients the static nonlinearities in a standardized way independent of the linear filter character so that ON, OFF, and ON-OFF cells can be easily identified and compared. For best results in this step only, the STA, STC, and STC-NC linear filters, as well as the stimulus vectors **s**_n_, were further reduced using a 300 µm×300 µm checker window. Because computing **L**_RSS_ is extremely computationally intensive, and because **L**_RSS_ did not vary significantly for each linear filter, we used an **L**_RSS_ that was averaged over 10 randomly chosen linear filters for the computation of all **N** vectors. Because the Gaussian distribution for our white noise was truncated at ∼2.5 standard deviations, the values of the **N** vector corresponding to **L**_RSS_ and **L**_STE_ inputs beyond 2.5 standard deviations of the **L**_RSS_ distribution were discarded. The ON/OFF/ON-OFF bias of the cell was then determined with the scalar value:
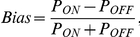
where *P_ON_* and *P_OFF_* represent integrals of the static nonlinearity over the positive or negative ranges, respectively. We labeled a cell OFF-center with 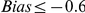, ON-OFF center with 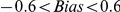, and ON-center with 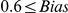.

### Measuring RF center size

RF center areas were computed from the STA and the STC-NC using two methods. In the first method, the single frame possessing the maximal deviation from the mean was isolated, and a bivariate Gaussian distribution was fit to this frame. The bivariate Gaussian function was described by:
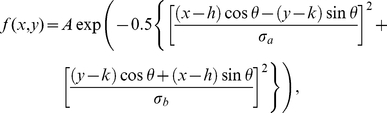
where A is the amplitude of the Gaussian, x and y are independent spatial coordinates, h and k are the x- and y-coordinate receptive field midpoints respectively, σ_a_ and σ_b_ are the standard deviations of the major and minor axes respectively, and θ is the angle between the major axis of the Gaussian and the x-axes of the coordinate system [53]. From the least squares fit of this distribution to the linear filter frame possessing maximal deviation from mean luminance, the RF center area within the 1σ ellipse of the Gaussian distribution was calculated according to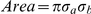. Because the STC-NC was windowed prior to computation, in order to improve fitting results, we loosely forced the Gaussian to zero at the edges of the STC-NC by framing it in a large surrounding zero-contrast border. A 6σ signal strength threshold was used to eliminate weakly responsive cells from further analysis.

The second method directly counted checkers in the frame of maximal deviation with amplitudes exceeding 0.35× the maximal magnitude. Although these two methods are not completely analogous, a comparison of the results shows that they are strongly correlated (p<0.001, R = 0.83; Fig. S6).

### Data Analysis for the spot stimulus

In a subset of the experiments, spot stimuli were applied in conjunction with Gaussian white noise. STA RF mapping was performed to identify the checker that most closely approximated the RF center. PSTHs were then generated to determine the cell's response to ON and OFF flashes in the single central checker. The peak firing rates in the first 500 ms following the ON and OFF flashes were used to calculate the Spot Bias with an equation analogous to that used in the calculation of the RDI:
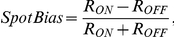
but now R_ON_ is the peak firing rate following the onset of the light spot, and R_OFF_ is the peak firing rate following the onset of the dark spot.

### Statistics

Comparison of distribution means was performed using the Student's *t*-test. Because the shape of the RF center size distributions departed significantly from Gaussian, a Wilcoxon rank sum test was used to compare RF center size medians. We also utilized the Analysis of Covariance technique (ANCOVA) to remove the effect of the confounding STC-NC signal strength prior to calculating the significance of differences in RF size due to grouping. The Kolmogorov-Smirnov (KS) test was used to compare the shapes of distributions of continuous-valued variables (such as STC-NC Bias), and a two sample χ^2^ test was used to compare distributions of categorical variables (such as ON, OFF, and ON-OFF classifications). Correlations were described using Pearson's linear correlation coefficient, and corresponding p-values were calculated using the Fisher transformation to map the correlation coefficient onto a t-statistic. Box plots utilized Matlab defaults, in which notch widths representing the 95% confidence interval for the median were calculated [54], and outliers were defined as data points lying 1.5 times the interquartile range beyond the upper or lower quartiles. To test for bimodality in the spike-triggered ensemble, the Hartigan and Hartigan Dip test was used, and p-values against the null hypothesis of unimodality were calculated by bootstrapping samples of the appropriate size drawn from a uniform distribution [55]–[58].

### Immunostaining of the mouse retina

Retinas were dissected and fixed in 4% (w/v) paraformaldehyde in 0.1 M phosphate-buffered saline (pH 7.4) at P16 [8]. Cryostat sections or whole-mounted retinas were prepared as described in Liu et al. (2007). The primary antibodies include anti-mouse Brn-3a (1∶100, Chemicon International) and anti-mouse SMI-32 (1∶1000, Sternberger Monoclonal Inc.). For confocal microscopy, images were captured with a Zeiss Pascal confocal microscope (Zeiss, Thornwood, NY) [8]. For cell counting, immuno-positive cells from 6–10 fields from each retina were counted and the average density was calculated in LSM5 Image browser (Zeiss) or ImageJ.

## Supporting Information

Figure S1 The STC-NC vector converges quickly. The STC-NC vector was calculated for a subset of cells at 100-spike intervals. The normalized estimated vector was projected onto the normalized final vector as a measure of error. The projection will yield 1 when the vectors are identical. (A–B) Plots of projection value against spike number for an ON (A) and an ON-OFF cell (B). The projection value reaches 0.8 by 2101 spikes for A and 1001 spikes for B. The projection value reaches 0.9 by 3501 spikes for A and 1801 for B. Both cells possess projection values >0.95 at half of their total spike counts, which was our criterion for convergence. Using a subset of only the fully converged cells (n = 10), we calculated that 1400±150 spikes are required for the projection to reach 0.8, and 2600±300 spikes are required to reach 0.9.
(0.10 MB TIF)

Figure S2 At P15, most cells have not developed mature light response characteristics in the WT retina. (A) 35% of spike trains recorded at P15 lacked a mappable RF. Such cells were unclassifiable and were discarded without further analysis. In fact, two of the five retinas at this age required that more than half of the spike trains be discarded. Because WT retinas at P15 yielded a smaller number of recorded cells per retina, possessed a larger percentage of unmappable, visually unresponsive cells, and demonstrated lower average STC-NC and STA signal response strengths, we concluded that the P15 retina was not yet mature. (B) The classification of ON, OFF, and ON-OFF cells may be particularly affected by the small total cell number as well as the large percentage of discarded cells. Nonetheless, with these disclaimers in mind, we used the STC-NC analysis to classify cells into ON, OFF, and ON-OFF categories at P15 (n = 5 retinas), and found that there was no significant difference between the WT cell distributions at P15 and P18 (χ^2^ p = 0.22). (C) The RF sizes for OFF and ON-OFF cells increased from P15 to P18, but for ON cells, the change was not significant. *: p<0.05; **: p<0.01 in Wilcoxon rank sum test. (D–F) Cumulative distributions of RF sizes for ON (D), OFF (E) and ON-OFF cells (F) in WT retinas from P15 to P18.
(0.18 MB TIF)

Figure S3 A comparison of alternative techniques for measuring RF center size supports measurement with a fitted bivariate Gaussian but suggests poor resolution of ON-OFF cell centers by the STA. Bar plot comparing RF center size measured with a fitted Gaussian using the STA and the STC-NC. The STA and STC-NC measured the same RF center size for ON (p = 0.35) and OFF (p = 0.71) cells, but the STC-NC measured a significantly increased RF center size for ON-OFF cells (p<0.001, Student's *t*-test).
(0.11 MB TIF)

Figure S4 Big checker and small checker stimuli exhibit a similar developmental trend in WT retinas. (A–B) two examples of an ON cell (A) and an ON-OFF cell (B) were exposed to both 60×60 µm and 100×100 µm checker stimuli for equal durations of time. The RF maps showed improved resolution with smaller checker sizes, but the response strength was also decreased with reduced spike counts and lower spike expectations. Moreover, for a large number of cells, we were only able to map the RFs with the 100×100 µm checkers because the smaller checkers did not elicit a strong enough response. (C) Despite these limitations, we observed a similar developmental trend from P18 to P25 with the two visual stimuli. Numbers of subtype cells were labeled in the bar graph. ***: P<0.001 in Wilcoxon rank sum test.
(0.26 MB TIF)

Figure S5 Diagram of the hexagonal (HexaMEA) and rectangular (RectMEA) layouts of the micro-electrode array (Multi-Channel Systems). HexaMEA has 60 electrodes with electrode spacing from 30, 60, to 90µm and electrode diameter from 10, 20, to 30µm. RectMEA has 60 electrodes with electrode spacing of 200µm and electrode diameter of 30µm.
(0.19 MB TIF)

Figure S6 Using the STC-NC, we plotted the 1σ RF center size determined by the fitted bivariate Gaussian against the RF center size determined by counting above-threshold squares. The best-fitting line forced through zero is plotted. Correlation coefficients (R) were calculated to measure the proportion of the data variance that is described by the best fitting line through zero. Squares were counted if they possessed a contrast that was more than 0.3 times the maximal contrast deviation in the frame. Because these two methods are not analogous, a correction was made to the RF center size determined by square counting. Briefly, we assumed that the RF was a radially symmetric bivariate Gaussian, and therefore the use of a threshold of 0.3 times the peak deviation is equivalent to measuring the area within the 1.5518σ contour. Given the square-count area and these assumptions, we can calculate σ and then the area within the 1σ contour. Importantly, this was a multiplicative correction, and it changed the slope, but not the correlation, which was very strong (R = 0.83).
(0.04 MB TIF)

Table S1 A summary of the advantages and disadvantages of each method.
(0.03 MB DOC)
