# Supplementary figures and images for: Non-Centered Spike-Triggered Covariance Analysis Reveals Neurotrophin-3 as a Developmental Regulator of Receptive Field Properties of ON-OFF Retinal Ganglion Cells
Source: PLoS Comput Biol. 2010 Oct 21;6(10):e1000967. doi: 10.1371/journal.pcbi.1000967 (PMC2958799; doi:10.1371/journal.pcbi.1000967)

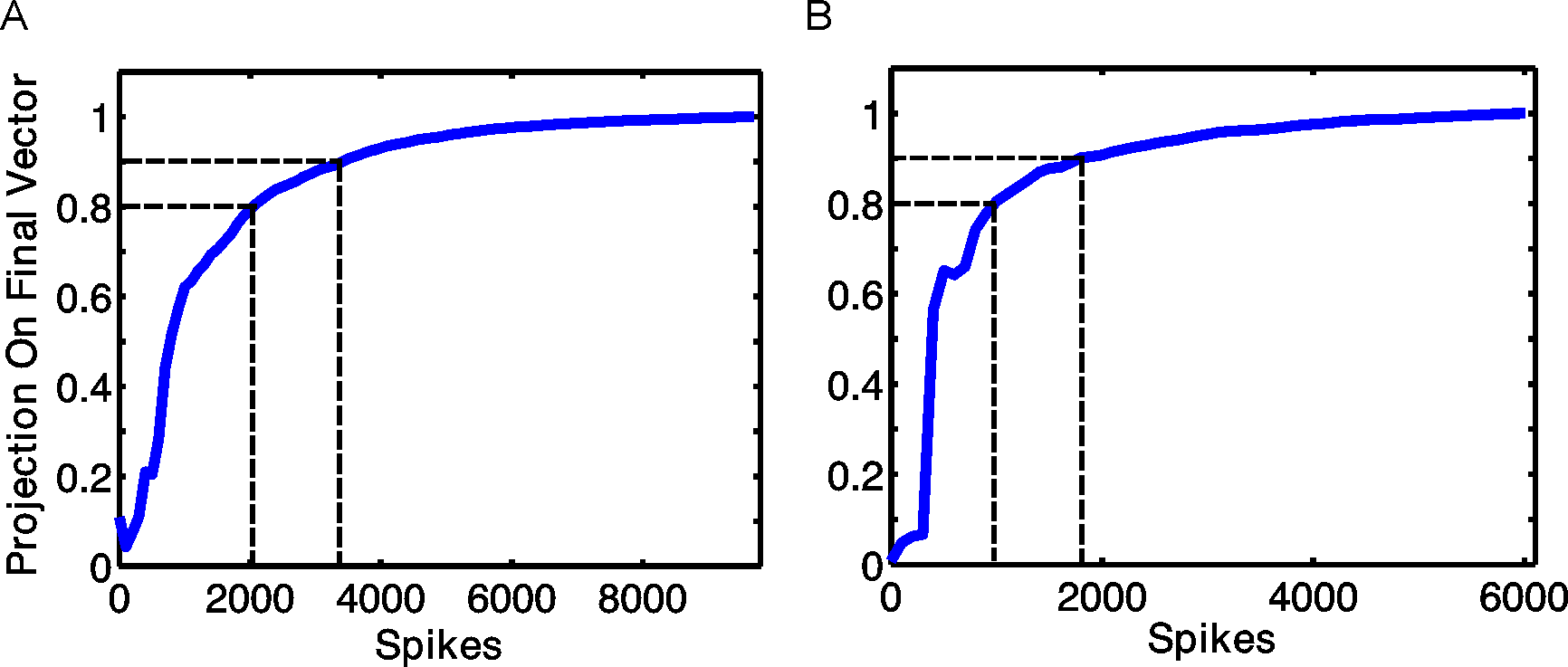

Supplement: Figure S1 — The STC-NC vector converges quickly. The STC-NC vector was calculated for a subset of cells at 100-spike intervals. The normalized estimated vector was projected onto the normalized final vector as a measure of error. The projection will yield 1 when the vectors are identical. (A–B) Plots of projection value against spike number for an ON (A) and an ON-OFF cell (B). The projection value reaches 0.8 by 2101 spikes for A and 1001 spikes for B. The projection value reaches 0.9 by 3501 spikes for A and 1801 for B. Both cells possess projection values >0.95 at half of their total spike counts, which was our criterion for convergence. Using a subset of only the fully converged cells (n = 10), we calculated that 1400±150 spikes are required for the projection to reach 0.8, and 2600±300 spikes are required to reach 0.9. (0.10 MB TIF) [file pcbi.1000967.s001.tif]

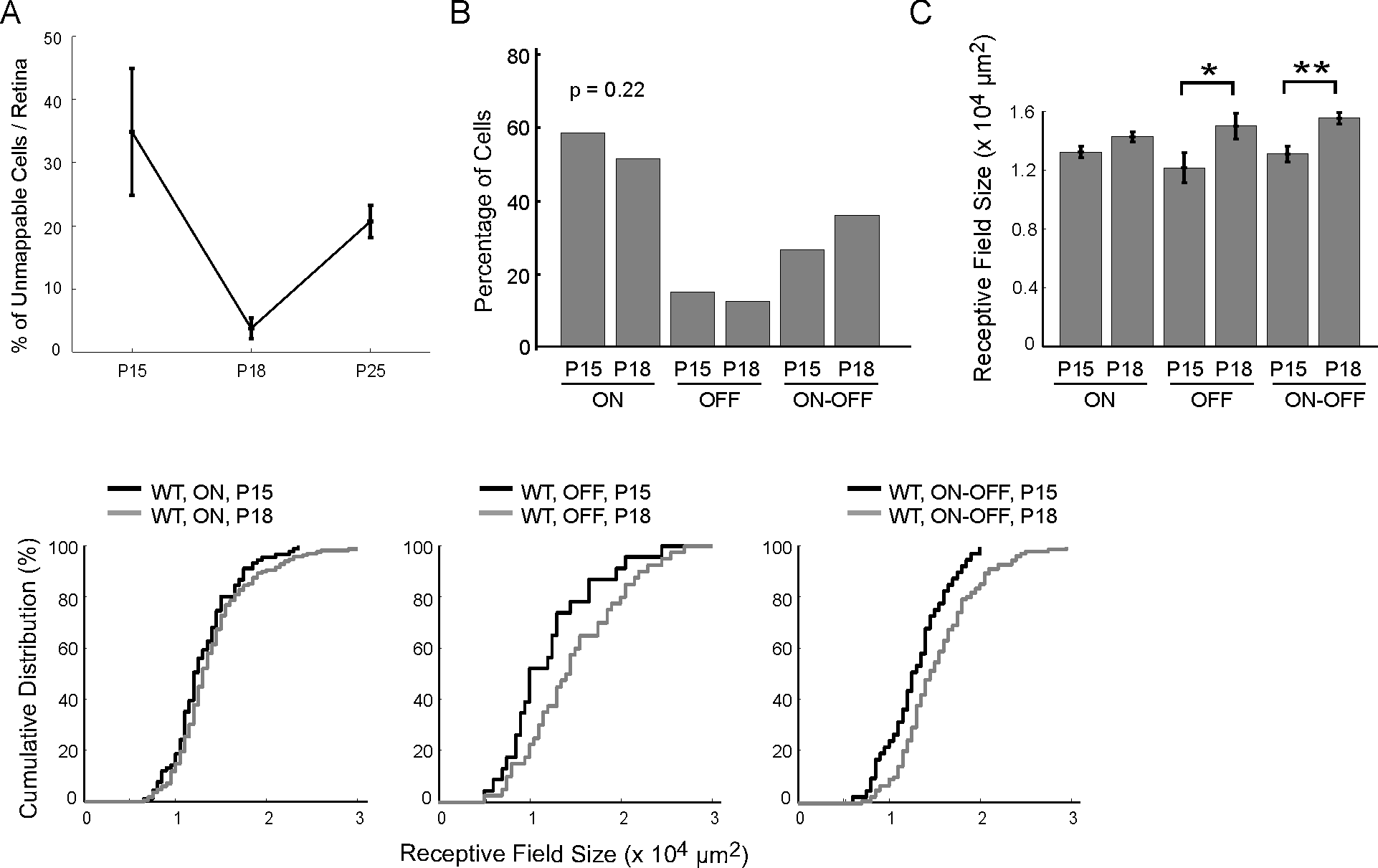

Supplement: Figure S2 — At P15, most cells have not developed mature light response characteristics in the WT retina. (A) 35% of spike trains recorded at P15 lacked a mappable RF. Such cells were unclassifiable and were discarded without further analysis. In fact, two of the five retinas at this age required that more than half of the spike trains be discarded. Because WT retinas at P15 yielded a smaller number of recorded cells per retina, possessed a larger percentage of unmappable, visually unresponsive cells, and demonstrated lower average STC-NC and STA signal response strengths, we concluded that the P15 retina was not yet mature. (B) The classification of ON, OFF, and ON-OFF cells may be particularly affected by the small total cell number as well as the large percentage of discarded cells. Nonetheless, with these disclaimers in mind, we used the STC-NC analysis to classify cells into ON, OFF, and ON-OFF categories at P15 (n = 5 retinas), and found that there was no significant difference between the WT cell distributions at P15 and P18 (χ2 p = 0.22). (C) The RF sizes for OFF and ON-OFF cells increased from P15 to P18, but for ON cells, the change was not significant. *: p<0.05; **: p<0.01 in Wilcoxon rank sum test. (D–F) Cumulative distributions of RF sizes for ON (D), OFF (E) and ON-OFF cells (F) in WT retinas from P15 to P18. (0.18 MB TIF) [file pcbi.1000967.s002.tif]

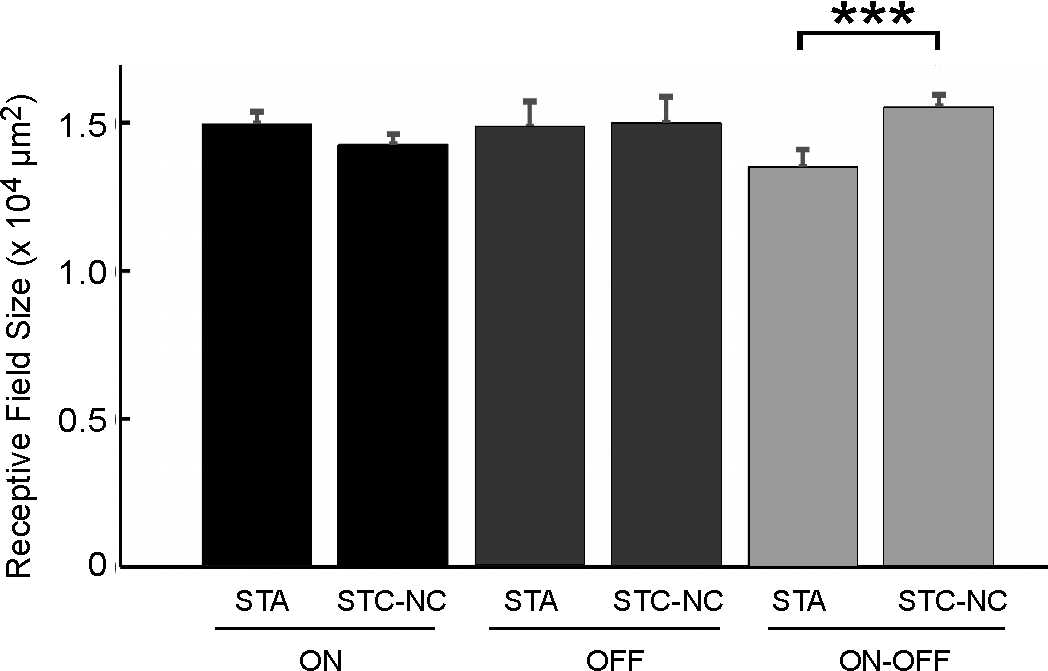

Supplement: Figure S3 — A comparison of alternative techniques for measuring RF center size supports measurement with a fitted bivariate Gaussian but suggests poor resolution of ON-OFF cell centers by the STA. Bar plot comparing RF center size measured with a fitted Gaussian using the STA and the STC-NC. The STA and STC-NC measured the same RF center size for ON (p = 0.35) and OFF (p = 0.71) cells, but the STC-NC measured a significantly increased RF center size for ON-OFF cells (p<0.001, Student's t-test). (0.11 MB TIF) [file pcbi.1000967.s003.tif]

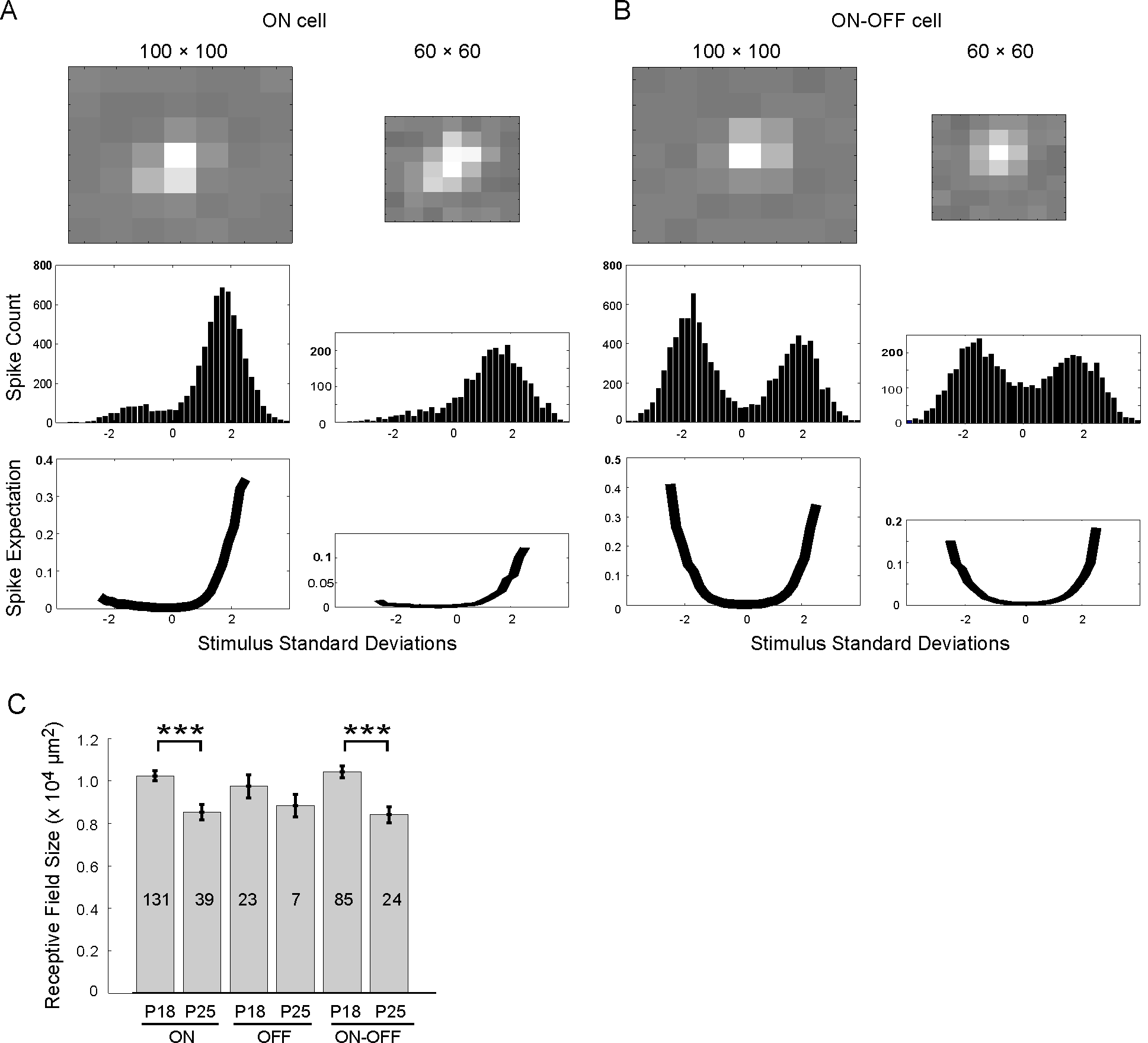

Supplement: Figure S4 — Big checker and small checker stimuli exhibit a similar developmental trend in WT retinas. (A–B) two examples of an ON cell (A) and an ON-OFF cell (B) were exposed to both 60×60 µm and 100×100 µm checker stimuli for equal durations of time. The RF maps showed improved resolution with smaller checker sizes, but the response strength was also decreased with reduced spike counts and lower spike expectations. Moreover, for a large number of cells, we were only able to map the RFs with the 100×100 µm checkers because the smaller checkers did not elicit a strong enough response. (C) Despite these limitations, we observed a similar developmental trend from P18 to P25 with the two visual stimuli. Numbers of subtype cells were labeled in the bar graph. ***: P<0.001 in Wilcoxon rank sum test. (0.26 MB TIF) [file pcbi.1000967.s004.tif]

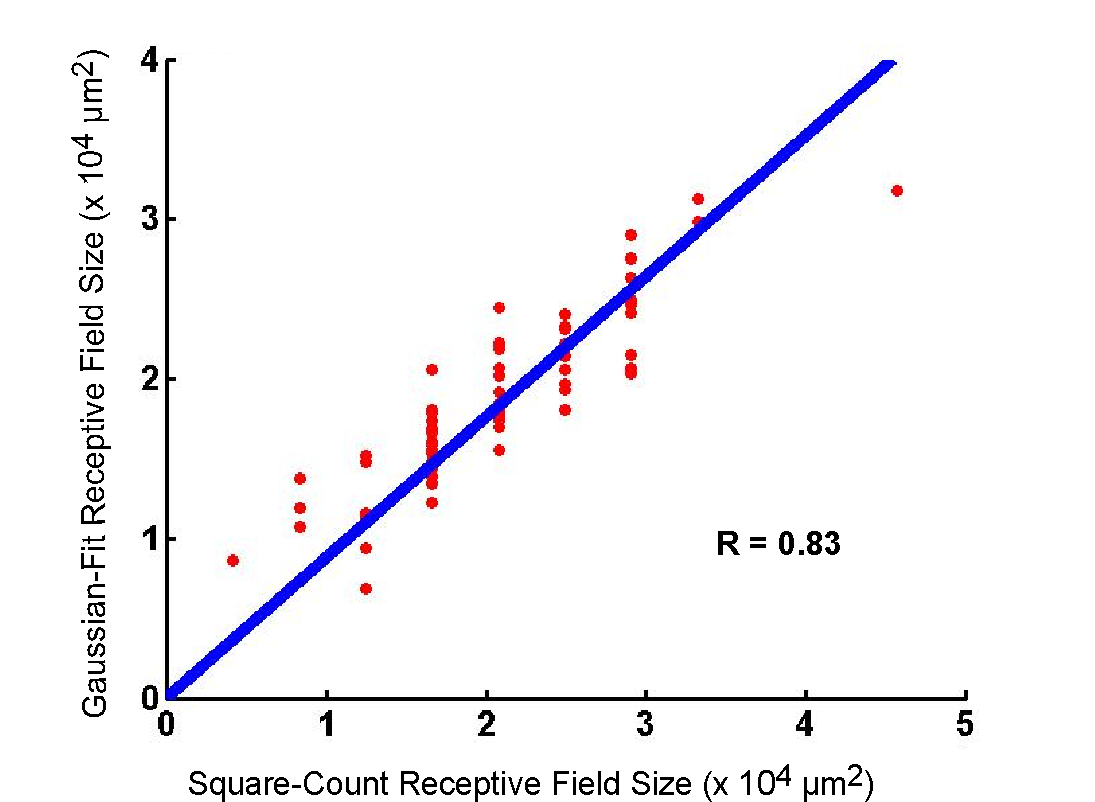

Supplement: Figure S5 — Diagram of the hexagonal (HexaMEA) and rectangular (RectMEA) layouts of the micro-electrode array (Multi-Channel Systems). HexaMEA has 60 electrodes with electrode spacing from 30, 60, to 90µm and electrode diameter from 10, 20, to 30µm. RectMEA has 60 electrodes with electrode spacing of 200µm and electrode diameter of 30µm. (0.19 MB TIF) [file pcbi.1000967.s005.tif]

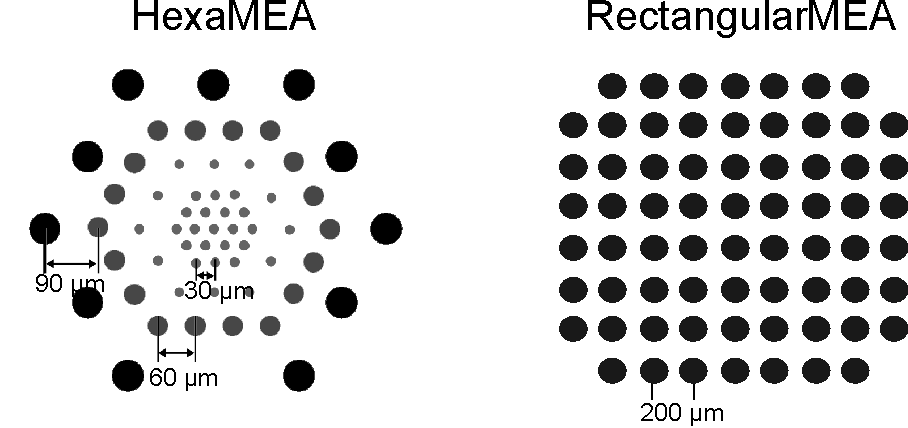

Supplement: Figure S6 — Using the STC-NC, we plotted the 1σ RF center size determined by the fitted bivariate Gaussian against the RF center size determined by counting above-threshold squares. The best-fitting line forced through zero is plotted. Correlation coefficients (R) were calculated to measure the proportion of the data variance that is described by the best fitting line through zero. Squares were counted if they possessed a contrast that was more than 0.3 times the maximal contrast deviation in the frame. Because these two methods are not analogous, a correction was made to the RF center size determined by square counting. Briefly, we assumed that the RF was a radially symmetric bivariate Gaussian, and therefore the use of a threshold of 0.3 times the peak deviation is equivalent to measuring the area within the 1.5518σ contour. Given the square-count area and these assumptions, we can calculate σ and then the area within the 1σ contour. Importantly, this was a multiplicative correction, and it changed the slope, but not the correlation, which was very strong (R = 0.83). (0.04 MB TIF) [file pcbi.1000967.s006.tif]
