# Supplementary material for: Non-Centered Spike-Triggered Covariance Analysis Reveals Neurotrophin-3 as a Developmental Regulator of Receptive Field Properties of ON-OFF Retinal Ganglion Cells
Source: PLoS Comput Biol. 2010 Oct 21;6(10):e1000967. doi: 10.1371/journal.pcbi.1000967 (PMC2958799; doi:10.1371/journal.pcbi.1000967)
Supplement: Table S1 — A summary of the advantages and disadvantages of each method. (0.03 MB DOC) [file pcbi.1000967.s007.doc]

**Table S1**

A summary of the advantages and disadvantages of each method

|  | Advantages | Disadvantages |
| --- | --- | --- |
| Full-field Flash | Stimulus easy to generate, robust cell responses | Inaccurate characterization of RF center properties |
| Spot Stimulus | Accurate characterization of RF center properties | Low throughput: One cell stimulated at a time |
| STA | High throughput: All cells stimulated simultaneously | Inaccurate characterization of ON-OFF RF center properties |
| Full STC | Full characterization of RF properties | Needs a very large number of spikes for each cell, and time-consuming for data analysis |
| STC-NC | Easy and accurate high-throughput classification of ON, OFF, and ON-OFF cells | Not a full characterization of RF properties |
